# Semi-Parametric Methods of Handling Missing Data in Mortal Cohorts under Non-Ignorable Missingness

**DOI:** 10.1111/biom.12891

**Published:** 2018-05-17

**Authors:** Lan Wen, Shaun R. Seaman

**Affiliations:** MRC Biostatistics Unit, University of Cambridge, IPH Forvie Site, Robinson Way, Cambridge CB2 0SR, U.K

**Keywords:** Dropout, Generalized estimating equation, Intermittent missing, Longitudinal data, Non-ignorable, Partly conditional inference, Sensitivity analysis

## Abstract

We propose semi-parametric methods to model cohort data where repeated outcomes may be missing due to death and non-ignorable dropout. Our focus is to obtain inference about the cohort composed of those who are still alive at any time point (partly conditional inference). We propose: i) an inverse probability weighted method that upweights observed subjects to represent subjects who are still alive but are not observed; ii) an outcome regression method that replaces missing outcomes of subjects who are alive with their conditional mean outcomes given past observed data; and iii) an augmented inverse probability method that combines the previous two methods and is double robust against model misspecification. These methods are described for both monotone and non-monotone missing data patterns, and are applied to a cohort of elderly adults from the Health and Retirement Study. Sensitivity analysis to departures from the assumption that missingness at some visit *t* is independent of the outcome at visit *t* given past observed data and time of death is used in the data application.

## 1 Introduction

In studies of the elderly, deaths occur frequently during follow-up and in most cases, truncate the outcome process. Several authors (e.g., Dufouil et al., 2004; Kurland et al., 2009; Seaman et al., 2016) have stressed the importance of distinguishing between outcomes that are missing due to dropout and those that are missing due to death. Otherwise we might find ourselves unintentionally defining post-death outcomes, which may be philosophically problematic. Some statistical methods do not make this distinction (e.g., linear mixed-effects models, LMM), and consequently estimate the mean or distribution of an outcome in the *whole* cohort, including subjects who are no longer alive. In doing so, these methods explicitly or implicitly impute post-death outcomes, as though the outcome process continued after death. Such methods are said to produce “immortal cohort inference” or “unconditional inference” (Dufouil et al., 2004). In contrast, methods that distinguish between dropout and death, and estimate the mean or distribution of the outcomes in the subjects who are alive provide “mortal cohort inference.”

Two forms of mortal cohort inference are “partly conditional inference” and inference about the average effect of an exposure on an outcome in the subpopulation who would survive regardless of their exposure status. The latter is known as the “survivor average causal effect” (SACE). In this article, we focus on partly conditional inference; the SACE is discussed in Web Appendix A. Partly conditional inference concerns the partly conditional mean, that is, the mean outcome (possibly conditional on covariates) at each time point in the subpopulation who are still alive at that time point. Estimating this mean for an outcome that is related to health-care need and how this mean depends on covariates can be useful for, for example, planning allocation of health-care resources, since it is this subpopulation who must be provided for.

The partly conditional mean can be estimated using Generalized Estimating Equations with an independence working correlation structure (IEE). IEE are valid if the missingness at a time point among those who are alive at that time point depends only on observed covariates. Kurland and Heagerty (2005) weaken this assumption by using inverse probability weighting (IPW) to weight observed outcomes by the inverse probability of observation among the subjects who are alive, given observed outcomes and covariates.

We are motivated by the Health and Retirement Study (HRS): a survey of adults 50 years or older in the United States. Data are collected every 2 years on aspects of life such as health, physical, and cognitive functioning, work, etc. In this article, we focus on data collected from 2004 (baseline) to 2012 and on adults 80 years or older at baseline. We aim to describe the average cognitive score of the subjects who are alive at each visit and to understand the factors associated with these subjects’ cognitive score while they were alive. One measure of cognitive function is total cognition score, which is the sum of total word recall and mental status summary scores, and has range 0–35.

Most statistical methods for missing data in cohort studies assume missing at random (MAR). The MAR assumption states that, conditional on observed data, missingness does not depend on the unobserved data (Seaman et al., 2013). However, Rotnitzky et al. (1998) and Scharfstein et al. (1999) (henceforth RRS) described semi-parametric methods for non-ignorable missing data, where missingness can depend on unobserved data. These articles deal with estimating the mean of a repeated outcome (possibly as a function of covariates) for monotone missing data, and rely on a selection bias function that quantifies the residual association between an outcome at a visit and the probability of observing this outcome after accounting for past outcomes and covariates. The parameter of this selection bias function is known as a sensitivity parameter. Vansteelandt et al. (2007) (henceforth VRR) proposed a class of semi-parametric models to handle non-monotone, non-ignorable missing data. Their (double-robust) method provides an estimator that is consistent and asymptotically normal when either a model for the probability of non-response given current outcome, past observed outcomes and covariates, or a model for the conditional mean of the missing outcome given past observed outcomes and covariates (or both) is correctly specified.

In a joint model for the outcomes and dropout, the sensitivity parameter can be estimated, but this estimate can be severely biased when the outcome submodel is misspecified (Robins and Rotnitzky, 1997). For this reason, RRS and VRR recommend assessing the effect on the estimate of interest by varying the selection bias function and/or sensitivity parameter.

RRS and VRR do not distinguish between death and other types of missingness. Wen et al. (2017) make this distinction and describe the assumptions of IPW for partly conditional inference, but only for monotone ignorably missing data. In this article, we adapt RRS and VRR’s methods to make partly conditional inference from monotone or non-monotone non-ignorably missing data caused by death, dropout and possibly return after dropout. In Section 2, we provide details about the motivating example. In Section 3, we define the assumptions for monotone missing data and describe our methods to make partly conditional inference. In Section 4, we define the assumptions for non-monotone missing data and adapt the semi-parametric methods from VRR to make partly conditional inference. In Section 5, we provide simulation studies to compare bias, efficiency, and coverage of the methods described in this article. In Section 6, we apply these methods to data from the HRS ageing study. All proofs are in the Web Appendix.

## 2 Motivation

Suppose there are *n* subjects in the study and *J* planned visits for each subject. Let *D_i_* be the last scheduled visit before subject *i* dies, and *A_it_* be his vital status at visit *t* (*t* = 1*, … , J*). Note that *A_it_* = 1 if and only if *D_i_* ≥ *t*, and that *D_i_* = *J* if subject *i* is still alive at the end of the study. Let *Y_it_* be the outcome at visit *t*, *Z_i_* be a vector of fully observed baseline covariates of interest, and *X*_*i*0_ be a vector that includes *Z_i_* and possibly other fully observed time-independent auxiliary variables. Let *X_it_* (*t* = 1*, … , J*) be a vector of auxiliary variables measured at time *t* (*X_it_* can be empty). The auxiliary variables are variables that are not of direct interest but may be predictive of missingness or missing outcomes. Let *R_it_* denote the response indicator (*R_it_* = 1 if *Y_it_* is observed, *R_it_* = 0 otherwise), and let ${\bar{R}}_{it}={\left(R_{i1},\ldots ,R_{it}\right)}^{T}.$ We define *A_i_*_0_ = 1 and $Y_{i0}=\emptyset .$ Henceforth, we omit subscripts *i* unless needed.

Our objective is to estimate the parameter *β* of a model for the mean outcome at each visit (possibly) given baseline covariates *Z* in those who are still alive at that visit: *μ_t_* = *μ_t_*(*Z*) = *E*(*Y_t_* | *Z, A_t_* = 1). In the HRS data analysis, we consider the model

(1) $$\mu_{t}=\beta_{0}+\beta_{t}{\text{year}}_{t}+\beta_{t^{2}}{\text{year}}_{t}^{2}+\beta_{\text{age}}\text{age}+\beta_{\text{sex}}\text{sex}+\beta_{\text{edu}}\text{edu}+\beta_{\text{tage}}{\text{year}}_{t}\cdot \text{age}+\beta_{\text{tsex}}{\text{year}}_{t}\cdot \text{sex}+\beta_{\text{tedu}}{\text{year}}_{t}\cdot \text{edu}$$

for the dependence of the expected cognitive function (*Y_t_*) at visit *t* on time (years from baseline, denoted year_*t*_), age at recruitment, sex (sex = 1 if female), years of education, and the interactions between time and age, sex, and education. Table 1 shows the results of applying LMM and IEE to the observed data. Since unhealthier subjects (those with lower, that is, worse cognitive function) are more likely to miss a visit than healthier subjects, the estimates from IEE are based on subjects who are healthier than average. On the other hand, the estimates from LMM are based on all subjects, and all the missing cognitive scores are implicitly imputed. If subjects are still alive, these imputed scores tend to be lower on average than in subjects who have not dropped out, otherwise they correspond to post-death outcomes. Hence, estimates from LMM suggest that the mean cognitive function declines more rapidly than do the estimates from IEE. However, LMM does not distinguish between death and other reasons for missingness, and IEE rely on strong assumptions about the missingness process. In the next two sections, we discuss methods that require weaker assumptions. Further results from this HRS example can be found in Section 6.

## 3 Non-Ignorable Monotone Missing Data in a Mortal Cohort

Under a monotone missing data pattern, when an outcome is missing at some visit *s* then all subsequent outcomes will also be missing (i.e., *R_t_* ≤ *R_s_*, for 1 ≤ *s* < *t* ≤ *J*). This type of missingness pattern occurs in cohort studies where subjects drop out but never return. Throughout this section, we let ${\bar{O}}_{t}=(X_{0},X_{1},\ldots ,X_{t},Y_{1},\ldots ,Y_{t})\;(t=1,\ldots ,J),\;\text{let}\;{\bar{O}}_{0}=X_{0},$ and assume the following (“Assumption 1”) holds:

$$P(R_{t}=1\;|\;R_{t-1}=1,{\bar{O}}_{t-1},Y_{t},A_{t}=1)>0,\;\forall t\;\text{with}\;\text{probability}\;1$$

We define “mortal-cohort non-future dependence (NFD)” as

$$P(R_{t}=0\;|\;R_{t-1}=1,{\bar{O}}_{t-1},Y_{t},\ldots ,Y_{D},D)=P(R_{t}=0\;|\;R_{t-1}=1,{\bar{O}}_{t-1},Y_{t},A_{t}=1),\forall t\leq D$$

Mortal-cohort NFD says that the probability of dropout at visit *t*, conditional on survival to visit *t*, can depend on past outcomes and the outcome at visit *t* but not on future outcomes or *D*. In ageing studies, it is not unlikely that someone’s mental state at a given time could affect their ability to participate in the study at that time. The rest of this section describes methods that yield consistent estimates under mortal-cohort NFD. The first method weights up outcomes from observed subjects to represent subjects who are still alive but have dropped out (IPW), the second method imputes pre-death missing outcomes (conditional mean outcome regression, CMOR), and the third method combines these two methods to offer double protection against model misspecification (Augmented IPW, AIPW).

### 3.1 Inverse Probability Weighting

Dufouil et al. (2004) first used IPW to make partly conditional inference for monotone missing data under non-ignorable dropout but did not describe the assumptions underlying their method. Below we clearly state the assumptions and the IPW estimating equations for making partly conditional inference. Let $\pi_{t}({\bar{O}}_{t-1},Y_{t};\alpha_{t},\gamma )$ be a model for $\pi_{t}({\bar{O}}_{t-1},Y_{t})=P(R_{t}=1\;|\;R_{t-1}=1,{\bar{O}}_{t-1},Y_{t},A_{t}=1)\;(t=1,\ldots J)$ with finite-dimensional parameters, *α_t_* and *γ*. For example, we could assume

(2) $$1-\pi_{t}({\bar{O}}_{t-1},Y_{t};\alpha_{t},\gamma )=\text{expit}(\alpha_{0t}+\alpha_{1t}Y_{t-1}+\alpha_{2t}X+\gamma Y_{t})$$

More generally, we assume the missingness model can be written as

(3) $$1-\pi_{t}({\bar{O}}_{t-1},Y_{t};\alpha_{t},\gamma )=\text{expit}\left\{h_{t}({\bar{O}}_{t-1};\alpha_{t})+q_{t}({\bar{O}}_{t-1},Y_{t};\gamma )\right\}$$

where $q_{t}({\bar{O}}_{t-1},Y_{t};\gamma )$ is a known selection bias function with parameter *γ* specified a priori, $h_{t}({\bar{O}}_{t-1};\alpha_{t})$ is a known function with unknown parameter *α_t_*, and expit(*a*) = { 1 + exp(−*a*)}^−1^. The function $q_{t}({\bar{O}}_{t-1},Y_{t};\gamma )$ describes the residual effect of the outcome at visit *t* on the probability of observing that outcome after adjusting for the observed data and missingness pattern up to visit *t* − 1. Note that if $q_{t}\left({\bar{O}}_{t-1},Y_{t};\gamma \right)=0,$ there is no residual dependence of the outcome at visit *t* on dropout. For monotone missing data this special case is referred to as unconditional-MAR in Wen et al. (2017), and details about its relationship with mortal cohort NFD can be found in Web Appendix H.

Let ${\hat{\alpha }}_{t}$ be the estimator of *α_t_* that solves

$$\sum_{i=1}^{n}Q_{it}(\alpha_{t})=\sum_{i=1}^{n}\frac{\phi_{t}({\bar{O}}_{i,t-1})A_{it}R_{i,t-1}}{\pi_{t}({\bar{O}}_{i,t-1},Y_{it};\alpha_{t},\gamma )}\times \left\{R_{it}-\pi_{t}({\bar{O}}_{i,t-1},Y_{it};\alpha_{t},\gamma )\right\}=0,\;\forall t$$

where $\phi_{t}({\bar{O}}_{t-1})$ is a function of ${\bar{O}}_{t-1}$ that has the same dimension as *α_t_*. For example, for model (2), $\phi_{t}({\bar{O}}_{t-1})$ could be (1*,Y_t_*_−1_*, X*)^*T*^. If mortal-cohort NFD holds, the selection bias function and the sensitivity parameter *γ* are correctly chosen, and the missingness models are correctly specified, then ${\hat{\alpha }}_{t}$ will be consistent.

Let *α* = (*α*_1_, … , *α_J_*) and $\hat{\alpha }=({\hat{\alpha }}_{1},\ldots ,{\hat{\alpha }}_{J}).$ The parameter *β* in the model of interest can be estimated by solving the following set of estimating equations:

(4) $$\sum_{i=1}^{n}\sum_{t=1}^{J}\left(\frac{\partial \mu_{it}}{\partial \beta }\right)\frac{A_{it}R_{it}(Y_{it}-\mu_{it})}{\lambda_{t}({\bar{O}}_{i,t-1},Y_{it};\hat{\alpha },\gamma )}=0$$

where $\lambda_{t}({\bar{O}}_{t-1},Y_{t};\hat{\alpha },\gamma )=\prod_{l=1}^{t}\pi_{l}({\bar{O}}_{l-1},Y_{l};{\hat{\alpha }}_{l},\gamma ).$. If mortal-cohort NFD holds, the selection bias function and the sensitivity parameter *γ* are correctly chosen, and the missingness models are correctly specified, then the estimator $\hat{\beta }$ that solves estimating equations (4) will be consistent.

### 3.2 Conditional Mean Outcome Regression

Here, we briefly outline the CMOR method; full details are in Web Appendix C.

Provided that Assumption 1 holds, equation (3) implies the following relation between the expected outcome (given history ${\bar{O}}_{t-1}$) at visit *t* in survivors who drop out just before visit *t* and in survivors who are observed at visit *t*.

(5) $$E(Y_{t}\;|\;{\bar{O}}_{t-1},R_{t-1}=1,R_{t}=0,A_{t}=1)=\frac{E\left[Y_{t}\text{exp}\left\{q_{t}({\bar{O}}_{t-1},Y_{t})\right\}\;|\;{\bar{O}}_{t-1},R_{t}=1,A_{t}=1\right]}{E\left[\text{exp}\left\{q_{t}({\bar{O}}_{t-1},Y_{t})\right\}\;|\;{\bar{O}}_{t-1},R_{t}=1,A_{t}=1\right]}$$

In particular, if $q_{t}\left({\bar{O}}_{t-1},Y_{t};\gamma \right)=0,$ then conditional on ${\bar{O}}_{t-1}$ and survival at visit *t*, subjects who drop out just before visit *t* have the same mean outcome at visit *t* as those who are observed at visit *t*. If $q_{t}({\bar{O}}_{t-1},Y_{t};\gamma )$ is an increasing (decreasing) function of *Y_t_*, then subjects who drop out just before visit *t* tend to have larger (smaller) *Y_t_* than those who are observed.

In the CMOR approach, the missing values of *Y_t_* in those who are alive at visit *t* but drop out just before visit *t* are imputed as $E(Y_{t}|{\bar{O}}_{t-1},R_{t-1}=1,R_{t}=0,A_{t}=1).$ Since this expectation is unknown, a model $m_{t}({\bar{O}}_{t-1};\theta_{t,t-1}),$ with parameters *θ*_*t,t*−1_, is specified for it (*t* = 1*, … , J*). By exploiting equation (5), *θ*_*t,t*−1_ can be estimated from the outcomes on subjects who are observed at visit *t*.

Next, provided Assumption 1 is true and mortal-cohort NFD holds, it can be shown that the mean outcome at visit *t* in survivors who drop out just before visit *t* − 1 is related to the mean outcome in survivors who are observed at visit *t* − 1 by:

(6) $$E(Y_{t}|{\bar{O}}_{t-2},R_{t-2}=1,R_{t-1}=0,A_{t}=1)=\frac{E_{Y_{t-1}}\left[E\left(Y_{t}\;|\;{\bar{O}}_{t-2},Y_{t-1},R_{t-1}=1,A_{t}=1\right)\;\text{exp}\{q_{t-1}({\bar{O}}_{t-2},Y_{t-1})\}\;|\;{\bar{O}}_{t-2},R_{t-1}=1,A_{t}=1\right]}{E\left[\text{exp}\left\{q_{t-1}({\bar{O}}_{t-2},Y_{t-1})\right\}\;|\;{\bar{O}}_{t-2},R_{t-1}=1,A_{t}=1\right]}.$$

Let $m_{t}({\bar{O}}_{t-2};\theta_{t,t-2})$ be a model for $E(Y_{t}|{\bar{O}}_{t-2},R_{t-2}=1,R_{t-1}=0,A_{t}=1)\;(t=2,\ldots ,J).$. By exploiting equation (6), *θ*_*t,t*−2_ can be estimated from the observed outcomes of survivors who are observed at visit *t*, and the already imputed outcomes of survivors who drop out just before visit *t*, that is, $m_{t}({\bar{O}}_{t-1};{\hat{\theta }}_{t,t-1}).$ The missing values of *Y_t_* in those who are alive at visit *t* but drop out just before visit *t* − 1 are then imputed as $m_{t}({\bar{O}}_{t-2};{\hat{\theta }}_{t,t-2}).$

The same idea is then used to impute missing *Y_t_* in subjects who are alive at visit *t* but drop out just before visit *t* − 2, then those who drop out just before visit *t* − 3, and so on. This method requires a model $m_{t}({\bar{O}}_{s};\theta_{t,s})$ for each $E(Y_{t}|{\bar{O}}_{s},R_{s}=1,R_{s+1}=0,A_{t}=1)(0\leq s<t\leq J).$ Note that post-death outcomes are not imputed.

Finally, having imputed all the missing pre-death outcomes, the parameter *β* in the model of interest is estimated by applying IEE to the imputed data set. If mortal-cohort NFD holds, the selection bias function and the sensitivity parameter *γ* are correctly chosen, and the regression models $m_{t}({\bar{O}}_{s};\theta_{t,s})$ are correctly specified, then this estimator of *β* is consistent.

### 3.3 Augmented Inverse Probability Weighting

We now propose augmented IPW (AIPW) estimating equations. These involve specifying a model for the probability of dropout and a regression model to fill in the missing outcomes with their expected values. The resulting estimator is doubly robust, that is, it is consistent when the missingness models are correctly specified at all visits, even when the regression models are not, and vice versa. Let *θ* = (*θ*_1,0_*, θ*_2,0_, …,*θ*_*J*,0_, *θ*_2,1_, *θ*_3,1_, …,*θ*_*J*,1_, *θ*_3,2_, …, *θ*_*J*,2_, …, *θ*_*J,J*−1_) and let $\hat{\theta }$ be the corresponding estimator. We utilize the IPW method described in Section 3.1 to model dropout and obtain $\hat{\alpha },$ and the CMOR method described in Section 3.2 to impute the missing outcome and obtain $\hat{\theta }$. Then we estimate *β* by solving

(7) $$\Psi \left(\hat{\alpha },\hat{\theta },\gamma ,\beta \right)=\sum_{i=1}^{n}\sum_{t=1}^{J}A_{it}\left(\frac{\partial \mu_{it}}{\partial \beta }\right)\times \left[\frac{R_{it}(Y_{it}-\mu_{it})}{\lambda_{t}({\bar{O}}_{i,t-1},Y_{it};\hat{\alpha },\gamma )}+\sum_{l=0}^{t-1}\frac{R_{il}}{\lambda_{l}({\bar{O}}_{i,l-1},Y_{il};\hat{\alpha },\gamma )}\left\{1-\frac{R_{i,l+1}}{\pi_{l+1}({\bar{O}}_{il},Y_{i,l+1};{\hat{\alpha }}_{l+1},\gamma )}\right\}\left\{m_{t}({\bar{O}}_{il};{\hat{\theta }}_{t,l})-\mu_{it}\right\}\right]=0$$

The resulting estimator $\hat{\beta }$ is consistent and asymptotically normally distributed if mortal-cohort NFD holds, the selection bias function and the sensitivity parameter *γ* are correctly chosen, and either the missingness models are correctly specified at all time points or the regression models are correctly specified at all time points. In Web Appendix G, we provide a formula for the asymptotic variance of $\hat{\beta }$ and a corresponding estimator. Note that if the missingness and regression models are misspecified, the variance estimator is still consistent, even though the point estimator $\hat{\beta }$ is, in general, not consistent.

### 3.4 Monotone Missing Data When D Is Known

*D* is likely to be known if individuals in a study are linked to a death registry. If *D* is known, then an option is to include it in the missingness or the regression models (or both for AIPW). If this is done, Assumption 1 should be modified to

$$P(R_{t}=1|R_{t-1}=1,{\bar{O}}_{t-1,}Y_{t},D,A_{t}=1)>0,\;\forall t\;\text{with probability1}$$

and mortal-cohort NFD modified to “fully conditional mortal-cohort NFD”:

$$P\left(R_{t}=0|R_{t-1}=1,{\bar{O}}_{t-1},Y_{t},\ldots ,Y_{D},D,A_{t}=1\right)=P\left(R_{t}=0|R_{t-1}=1,{\bar{O}}_{t-1},Y_{t},D,A_{t}=1\right)$$

In a sensitivity analysis, we can quantify the effect of perturbations to the assumption that $q_{t}\left({\bar{O}}_{t-1},Y_{t};\gamma \right)=0$ (i.e., the assumption that ${\bar{O}}_{t-1}$ includes all the variables that explain missingness at visit *t*). This assumption is made more plausible if we include *D* in the missingness or regression model, as people may be more likely to drop out if they are near death. Note that $P(R_{t}=0|R_{t-1}=1,{\bar{O}}_{t-1},Y_{t},D,A_{t}=1)=P(R_{t}=0|R_{t-1}=1,{\bar{O}}_{t-1},D,A_{t}=1)$ is referred to as fully conditional-MAR in Wen et al. (2017).

When *D* is included in both missingness and regression models and $q_{t}\left({\bar{O}}_{t-1},Y_{t};\gamma \right)=0,$ the AIPW estimator that solves equations (7) is equivalent to the AIPW estimator given in Wen et al. (2017) (see Web Appendix F for proof).

## 4 Non-Ignorable Non-Monotone Missing Data in a Mortal Cohort

Non-monotone missingness occurs when a subject who misses a scheduled visit may return at a later visit. In this section, we give estimators for non-ignorable non-monotone missing data by adapting the methods from VRR to make partly conditional inference. We redefine ${\bar{O}}_{t}$ as ${\bar{O}}_{t}=(X_{0},R_{1},R_{1}X_{1},R_{1}Y_{1},\ldots ,R_{t},R_{t}X_{t},R_{t}Y_{t}),$ and make “Assumption 2”:

$$P\left(R_{t}=1|{\bar{O}}_{t-1},Y_{t},A_{t}=1\right)>0,\;\forall t\;\text{with probability one}$$

Let $\lambda_{t}\left({\bar{O}}_{t-1},Y_{t};\alpha_{t},\gamma \right)$ be a model for $\lambda_{t}\left({\bar{O}}_{t-1},Y_{t}\right)=P\left(R_{t}=1|{\bar{O}}_{t-1},Y_{t},A_{t}=1\right)$ with finite dimensional parameters *α_t_* and *γ*. The general functional form for $\lambda_{t}\left({\bar{O}}_{t-1},Y_{t};\alpha_{t},\gamma \right)$ is given by equation (3), but with $\pi_{t}\left({\bar{O}}_{t-1},Y_{t};\alpha_{t},\gamma \right)$ replaced by $\lambda_{t}\left({\bar{O}}_{t-1},Y_{t};\alpha_{t},\gamma \right)$. Note that if we assume that $q_{t}\left({\bar{O}}_{t-1},Y_{t};\gamma \right)=0,$ we obtain the “mortal-cohort sequential explainability” assumption:

(8) $$P\left(R_{t}=1\;|\;{\bar{O}}_{t-1},Y_{t},A_{t}=1\right)=P\left(R_{t}=1\;|\;{\bar{O}}_{t-1},A_{t}=1\right),\;\forall t$$

which correspond to sequential explainability (Vansteelandt et al., 2007)—the assumption that *R_t_* is independent of *Y_t_* given ${\bar{O}}_{t-1}$—conditional on subjects being alive.

### 4.1 Inverse Probability Weighting

If Assumption 2 holds, the selection bias function and the sensitivity parameter are correctly chosen, and the model for $\lambda_{t}({\bar{O}}_{t-1},Y_{t})$ is correctly specified, then the estimator ${\hat{\alpha }}_{t}$ that solves

$$\sum_{i=1}^{n}\frac{\phi_{t}\left({\bar{O}}_{i,t-1}\right)A_{it}}{\lambda_{t}\left({\bar{O}}_{i,t-1},Y_{it};\alpha_{t},\gamma \right)}\{R_{it}-\lambda_{t}\left({\bar{O}}_{i,t-1},Y_{it};\alpha_{t},\gamma \right)\}=0,\;\forall t$$

where $\phi_{t}\left({\bar{O}}_{t-1}\right)$ is a function ${\bar{O}}_{t-1}$ that has the same dimension as *α_t_*, is consistent. Consequently, the estimator $\hat{\beta }$ that solves equations (4) is consistent.

### 4.2 Conditional Mean Outcome Regression

As in Section 3.2, we can relate the expected outcome at visit *t* given ${\bar{O}}_{t-1}$ in survivors who are not observed at visit *t* to the expected outcome in survivors who are observed at visit *t*:

$$E\left(Y_{t}|{\bar{O}}_{t-1,}R_{t}=0,A_{t}=1\right)=\frac{E[Y_{t}\text{exp}\{q_{t}\left({\bar{O}}_{t-1},Y_{t}\right)\}|{\bar{O}}_{t-1},R_{t}=1,A_{t}=1]}{E\left[\text{exp}\{q_{t}\left({\bar{O}}_{t-1},Y_{t}\right)\}|{\bar{O}}_{t-1},R_{t}=1,A_{t}=1\right]}$$

Let $m_{t}\left({\bar{O}}_{t-1};\theta_{t}\right)$ be a regression model for $m_{t}\left({\bar{O}}_{t-1}\right)=E\left(Y_{t}\;|\;{\bar{O}}_{t-1},R_{t}=0,A_{t}=1\right)$ with finite dimensional parameter *θ_t_*. If the selection bias function and the sensitivity parameter are correctly chosen, and the model for $m_{t}\left({\bar{O}}_{t-1}\right)$ is correctly specified, then the estimator ${\hat{\theta }}_{t}$ that solves $\sum_{i=1}^{n}A_{it}R_{it}\;\text{exp}\left\{q_{t}\left({\bar{O}}_{i,t-1},Y_{it}\right)\right\}$ $\left\{Y_{it}-m_{t}\left({\bar{O}}_{i,t-1};\theta_{t}\right)\right\}d_{t}\left({\bar{O}}_{i,t-1}\right)=0,$ where $d_{t}\left({\bar{O}}_{t-1}\right)$ is a function of ${\bar{O}}_{t-1}$ that has the same dimension as *θ_t_*, is consistent. Replacing the missing pre-death outcomes with their imputed values estimated from $m_{t}\left({\bar{O}}_{t-1};{\hat{\theta }}_{t}\right)$ and analysing the imputed data set using IEE will then give consistent estimates of *β*.

### 4.3 Augmented Inverse Probability Weighting

The AIPW estimators in VRR are attractive because the estimates of *β* are consistent as long as one of missingness model and regression model is correctly specified at each visit (i.e., if, for each *t*, either $h_{t}\left({\bar{O}}_{t-1};\alpha_{t}\right)$ or $m_{t}\left({\bar{O}}_{t-1};\theta_{t}\right)$ is correctly specified) and the selection bias function and the sensitivity parameter are correct. To make partly conditional inference, we modify their doubly robust estimating equations to be the following:

$$\sum_{i=1}^{n}\sum_{t=1}^{J}A_{it}\frac{\partial \mu_{it}}{\partial \beta }\left[\frac{R_{it}}{\lambda_{t}\left({\bar{O}}_{i,t-1},Y_{it};{\hat{\alpha }}_{t},\gamma \right)}\left(Y_{it}-\mu_{it}\right)+\left\{1-\frac{R_{it}}{\lambda_{t}\left({\bar{O}}_{i,t-1},Y_{it};{\hat{\alpha }}_{t},\gamma \right)}\right\}\left\{m_{t}\left({\bar{O}}_{i,t-1};{\hat{\theta }}_{t}\right)-\mu_{it}\right\}\right]=0$$

Note that, whereas the AIPW estimator for monotone missing data in Section 3.3 gives consistent estimation if the missingness models are correctly specified at all time points or the regression models are correctly specified at all time points, this AIPW estimator for non-monotone missing data gives consistent estimation if at each time point, either the missingness model or the regression model is correctly specified.

### 4.4 Non-Monotone Missing Data When D Is Known

If *D* is known for all subjects in a study, it can be included in the missingness and/or the regression models. If this is done, Assumption 2 should be modified to

(9) $$P\left(R_{t}=1\;|\;{\bar{O}}_{t-1},Y_{t},D,A_{t}=1\right)>0,\;\forall t\;\text{with probability}\;1$$

We define “fully conditional mortal-cohort sequential explainability” as the following modified version of equation (8):

(10) $$P\left(R_{t}=1\;|\;{\bar{O}}_{t-1},Y_{t},D,A_{t}=1\right)=P\left(R_{t}=1\;|\;{\bar{O}}_{t-1},D,A_{t}=1\right),\;\forall t$$

As discussed in Section 3.4, we could include *D* in the missingness or regression model to make fully conditional mortal-cohort sequential explainability more plausible.

## 5 Simulation Studies

We conducted two simulation studies to compare the methods. In each simulated data set, approximately 30% of outcomes were missing due to death, and approximately 25% of outcomes in those who are alive at each visit were missing. There were *J* = 5 biennial scheduled visits, and *P*(*R*_1_ = *A*_1_ = 1) = 1. Each simulation study was based on 1000 simulated data sets of sample size *n* = 500, and our aim is to estimate

$$E\left(Y_{t}\;|\;A_{t}=1\right)=\beta_{1}+\beta_{2}\text{I}\left(t=2\right)+\beta_{3}\text{I}\left(t=3\right)+\beta_{4}\text{I}\left(t=4\right)+\beta_{5}\text{I}\left(t=5\right)$$

In simulation one, data were monotone missing (“monotone study”) and in simulation two, data were non-monotone missing (“non-monotone study”).

*X* is a baseline variable with *X* ~ Normal(2, 4). Let *U* = |*X*|^1.5^. In both studies, the outcome *Y*_1_ was simulated from *Y*_1_ | *X* ~ Normal(5 − 0.1*U,*1), and vital status at each visit (*t* ≥ 2) was generated from logistic regression model, $P\left(A_{t}=1\;|\;A_{t-1}=1,{\bar{Y}}_{t-1},X\right)=\text{expit}\left(1.5+0.15Y_{t-1}-0.05U\right).$ For *t* ≥ 2, outcome *Y_t_* in the monotone study was simulated from $Y_{t}|{\bar{O}}_{t-1},A_{t}=1∼\text{Normal}\left(5-0.2\cdot {\text{year}}_{t}-0.1U+0.05Y_{t-1},1\right),$ and missingness was generated from $P\left(R_{t}=0|R_{t-1}=1,{\bar{O}}_{t-1},Y_{t},A_{t}=1\right)=\text{expit}\left(-0.75-0.175Y_{t-1}+0.1U-0.2Y_{t}\right).$

For *t* ≥ 2, outcome *Y_t_* in the non-monotone study was simulated from $Y_{t}\;|\;{\bar{Y}}_{t-1},X,R_{t-1}=r,{\bar{R}}_{t-2},A_{t}=1∼\text{Normal}\left(5+\alpha_{r}\cdot {\text{year}}_{t}-0.1U+0.05Y_{t-1},1\right),$ where *α*_0_ = −0.4 and *α*_1_ = −0.2; missingness at each visit was generated from $P\left(R_{t}=0\;|\;{\bar{O}}_{t-1},Y_{t},A_{t}=1\right)=\text{expit}\left(0.1-0.175Y_{t-1}^{†}+0.1U-0.2Y_{t}\right),$ where $Y_{t-1}^{†}=Y_{t-1}$ if *Y*_*t*−1_ is observed and 0 otherwise.

Note that in both simulations, $q_{t}\left({\bar{O}}_{t-1},Y_{t};\gamma \right)=\gamma Y_{t}$ with *γ* =−0.2. In the monotone study, the correct missingness and regression models include *Y*_*t*−1_ and *U*. In the non-monotone study, the correct missingness model includes $Y_{t-1}^{†}$ and *U*, and the correct regression model includes *R*_*t*−1_, $Y_{t-1}^{†}$ and *U*. We show the double robustness of the proposed AIPW method in the monotone study by replacing *U* by *X* in the missingness or regression models at all visits, and in the non-monotone study by omitting *U* from the regression model at visit 4 and from the missingness model at visit 5.

Table 2 shows the bias, empirical standard error and coverage of 95% confidence intervals from IPW, CMOR, and AIPW in the monotone study. Under correctly specified missingness and regression models, the parameter estimates from all three methods are nearly unbiased. When the regression models are correctly specified and the missingness models are not, AIPW provides nearly unbiased parameter estimates but IPW does not. Conversely, when the missingness models are correctly specified and the regression models are not, AIPW is nearly unbiased but CMOR is not. In our simulation, AIPW is at least as efficient as IPW when both the missingness and regression models are correctly specified.

Table 3 shows the biases, empirical standard errors, and coverages in the non-monotone study. Under correctly specified missingness and regression models, the estimates of *β*_4_ and *β*_5_ from all three methods are nearly unbiased. The IPW estimator of *β*_5_ is biased when the missingness model at visit 5 is misspecified, and similarly the CMOR estimator of *β*_4_ is biased when the regression model at visit 4 is misspecified. In contrast, the AIPW estimators of *β*_4_ and *β*_5_ are nearly unbiased when one of the missingness or regression models is misspecified, but not both. Again AIPW is at least as efficient as IPW when both models are correctly specified. Table 4 shows a sensitivity analysis in which *γ* is varied from 0 to −0.5. As expected, the results show that as the assumed value of *γ* deviates from its true value (−0.2), the bias increases (for all three methods).

In general, the variances of *β*_4_ and *β*_5_ are slightly underestimated by all three methods, due to slow convergence to the normal limiting distribution. This is reflected in the slightly lower coverage probabilities for *β*_4_ and *β*_5_. We see better results, in general, when *n* gets larger. In the non-monotone study, for example, the coverage probability for *β*_5_ in the IPW method was 91.6% when *n* = 500, but was 94.1% when *n* = 1000. Previous articles such as Shardell and Miller (2008) have also noted the robust variance estimates lead to undercoverage of confidence intervals at small sample sizes and that bootstrap provides better variance estimates. For this reason, we recommend using bootstrap to calculate standard error, as is done in the following analysis of the HRS data.

## 6 Application of Methods to HRS

The aim in this illustrative example is to understand how mean cognitive function given survival changes over time and how it depends on age, sex, and education. Researchers have previously classified adults older than 80 or 85 as the “oldest old” in various cohort studies (e.g., the Origins of Variance in the Old-Old, the English Longitudinal Study of Ageing, and the Survey of Health, Ageing and Retirement in Europe studies), and many have emphasized the importance of studying this group of subjects. As described by the National Institute of Ageing: “Over time, more older people survive to even more advanced ages. […] Because of chronic disease, the oldest old have the highest population levels of disability that require long-term care. They consume public resources disproportionately as well.” Hence, it is important to describe how cognitive function changes in the oldest old, as it is indicative of mental disability and therefore affects care requirements. Being able to estimate average cognitive function is important for making decisions about the allocation of care resources.

We focus on adults who were 80 years or older in 2004, and the model of interest is that given by equation (1). We exclude subjects who entered the study after 2004 or died before 2004 or had missing cognitive scores at all five visits. With the exception of 11 subjects, vital status is known at each scheduled visit time up to the end of the study. After additionally removing these 11 subjects, the number of subjects in our sample is 2616. 33% of the cognitive scores are missing due to death and 15% are missing due to other reasons. Among the outcomes of those who are alive at each visit, 3% are intermittent missing. To analyze these non-monotone missing data, we use the methods from Section 4. The first class of selection bias functions that we consider is {*γY_t_* : *γ* ∈ ℝ}. It is plausible that the residual association between *R_t_* and *Y_t_* after adjusting for ${\bar{O}}_{t-1}$ is different in subjects who were observed at the last visit than in those who were not, since ${\bar{O}}_{t-1}$ includes *Y*_*t*−1_ for the first group but not for the second group. Hence we consider a second class of selection bias function: {*γ*_1_*R*_*t*−1_*Y_t_* + *γ*_2_(1 − *R*_*t*−1_)*Y_t_* : *γ*_1_, *γ*_2_ ∈ ℝ}.

We first consider the case where *γ* = 0 (or *γ*_1_ = *γ*_2_ = 0). This corresponds to the assumption that ${\bar{O}}_{t-1}$ sufficiently explains the reasons for missingness at visit *t*. Including *D* in the missingness or regression model makes this assumption more plausible in the HRS data, because people were more likely to miss a visit when they were near death. Hence, we let fully conditional mortal-cohort sequential explainability be a benchmark assumption, and perform sensitivity analysis to determine if the *β* parameter estimates are robust to deviations from this benchmark. The missingness and regression models for visit *t* include sex, education, *R*_*t*−1_, observed *Y*_*t*−1_ (i.e., *R*_*t*−1_*Y*_*t*−1_), baseline age, and *D*.

### 6.1 First Class of Selection Bias Function: {*γY_t_* : *γ* ∈ℝ}

Here, *γ* is the log odds ratio of missing a visit at *t* for subjects whose *Y_t_* = *y* compared to missing a visit at *t* for subjects whose *Y_t_* = *y* − 1, with ${\bar{O}}_{t-1}$ and *D* held constant:

$$\text{exp}\left(\gamma \right)=\frac{P\left(R_{t}=0|{\bar{O}}_{t-1},Y_{t}=y,D,A_{t}=1\right)}{P\left(R_{t}=1|{\bar{O}}_{t-1},Y_{t}=y,D,A_{t}=1\right)}/\frac{P\left(R_{t}=0|{\bar{O}}_{t-1},Y_{t}=y-1,D,A_{t}=1\right)}{P\left(R_{t}=1|{\bar{O}}_{t-1},Y_{t}=y-1,D,A_{t}=1\right)}$$

Negative values of *γ* imply that those with lower cognitive scores are more likely to miss a visit than those with higher cognitive scores. We assume *γ* ≤ 0, because people with lower cognitive scores are likely to be more frail than people with higher cognitive scores and therefore more likely to miss a visit. As *γ* becomes increasingly negative, we would expect to see a decrease in the proportion of higher cognitive scores in the missing data, so that for extreme negative values of *γ*, all missing cognitive scores would be low. We consider a range of values for *γ* of [0, −0.3]. The rationale for this range is that in an exploratory analysis conditioning on sex, education, *R*_*t*−1_, observed *Y*_*t*−2_ (i.e., *R*_*t*−2_*Y*_*t*−2_), baseline age and *D*, the estimated log odds of missing a visit at times 4, 6, and 8 (i.e., visits 3, 4, and 5) per unit increase in observed *Y*_*t*−1_ were respectively −0.157, −0.166, and −0.145. Hence, we would also expect that those with worse cognitive function at visit *t* are more likely to be missing at visit *t* than those with better cognitive function at visit *t*. However, we also expect a stronger dependence of missingness at visit *t* on *Y_t_* than on *Y*_*t*−1_. Therefore we allowed *γ* to be as low as −0.3, which is almost twice as big as the associations between the log odds of missingness at visit *t* and *Y*_*t*−1_. *γ* = −0.3 indicates that the odds of missing visit *t* is reduced by 26% if *Y_t_* = *y* instead of *Y_t_* = *y* − 1, with all other variables held constant. In the Web Appendix I, we show results for more extreme values of *γ* (up to −0.70).

### 6.2 Second Class of Selection Bias Function: {*γ*_1_*R*_*t*−1_*Y_t_* + *γ*_2_(1 − *R*_*t*−1_)*Y_t_* : *γ*_1_*, γ*_2_ ∈ ℝ}

Here, *γ*_1_ (respectively, *γ*_2_) is the log odds ratio of missing a visit at *t* for subjects whose *Y_t_* = *y* and *R*_*t*−1_ = 1 (*R*_*t*−1_ = 0) compared to subjects whose *Y_t_* = *y* − 1 and *R*_*t*−1_ = 1 (*R*_*t*−1_ = 0), with *Ō*_*t*−1_ and *D* held constant:

$$\text{exp}\{\gamma_{1}R_{t-1}+\gamma_{2}\left(1-R_{t-1}\right)\}=\frac{P\left(R_{t}=0|{\bar{O}}_{t-1},Y_{t}=y,D,A_{t}=1\right)}{P\left(R_{t}=1|{\bar{O}}_{t-1},Y_{t}=y,D,A_{t}=1\right)}/\frac{P\left(R_{t}=0|{\bar{O}}_{t-1},Y_{t}=y-1,D,A_{t}=1\right)}{P\left(R_{t}=1|{\bar{O}}_{t-1},Y_{t}=y-1,D,A_{t}=1\right)}$$

Since *Y*_*t*−1_ and *Y_t_* are associated, when *Y_t_* is observed (i.e., *R_t_* = 1) one can think of *Y*_*t*−1_ as “absorbing” part of the effect of *Y_t_* on *R_t_*. So, when *R*_*t*−1_ = 0, the residual effect of *Y_t_* on *R_t_* may be greater than when *R*_*t*−1_ = 1. Thus, we assume *γ*_2_ ≤ *γ*_1_ ≤ 0 and consider *γ*_1_ = {−0.2, −0.25, −0.3} and *γ*_2_ = *cγ*_1_, where *c* = {1.25, 1.5, 2}.

### 6.3 Results

The parameter estimates and standard errors from the first selection bias function are shown in Table 5. In general, the parameters associated with *t* (*β_t_*, *β*_tage_, *β*_tsex_) were sensitive to the choice of *γ*. First, ${\hat{\beta }}_{t}$ ranged from −0.125 (p = 0.32; *γ* = 0) to −0.245 (p = 0.05; *γ* = −0.3) in IPW, and from −0.118 (p = 0.35; *γ* = 0) to −0.208 (p = 0.09; *γ* = −0.3) in AIPW. Hence in IPW and AIPW, when the association between *R_t_* and *Y_t_* given ${\bar{O}}_{t-1}$ and *D* is stronger, the downward linear trend in the mean is bigger. Second, ${\hat{\beta }}_{\text{tage}}$ ranged from −0.030 (p < 0.001; *γ* = 0) to −0.018 (p = 0.08; *γ* = −0.3) in IPW, and from −0.026 (p = 0.002; *γ* = 0) to −0.018 (p = 0.03; *γ* = −0.3) in AIPW. Hence in IPW and AIPW, when the association between *R_t_* and *Y_t_* given ${\bar{O}}_{t-1}$ and *D* is stronger, the difference between the rates of change over time in mean outcome given survival in old and young subjects is smaller. Third, ${\hat{\beta }}_{\text{tsex}}$ ranged from −0.101 (p = 0.11; *γ* = 0) to −0.206 (p = 0.001; *γ* = −0.3) in IPW, from −0.037 (p = 0.43; *γ* = 0) to −0.100 (p = 0.09; *γ* = −0.3) in CMOR, and from −0.105 (p = 0.08; *γ* = 0) to −0.157 (p = 0.006; *γ* = −0.3) in AIPW. Hence, when the association between *R_t_* and *Y_t_* given ${\bar{O}}_{t-1}$ and *D* is stronger, the difference between the rates of change over time in mean outcome given survival in males and females is bigger.

Table 5 shows that for values of *γ* between −0.2 and −0.3, qualitative conclusions from IPW, CMOR, and AIPW did not differ much. AIPW (e.g., when *γ* = −0.25) suggests that, controlling for other variables, i) the older a person is at recruitment, the worse their initial cognitive function is $\left({\hat{\beta }}_{age}=-0.325,\;\text{p}<0.001\right);$ ii) the more education a person has, the better their initial cognitive function is $\left({\hat{\beta }}_{edu}=0.730,\;\text{p}<0.001\right);$ and iii) the change over time in mean cognitive function given survival is greater in the group who are older at recruitment or are female than in the group who are younger $\left({\hat{\beta }}_{\text{tage}}=-0.018,\;\text{p}=0.03\right)$ or male $\left({\hat{\beta }}_{\text{tsex}}=0.153,\;\text{p}=0.006\right).$

In Web Appendix I, Table 2 shows results for more extreme values of *γ*, and Table 3 shows results from using the second selection bias function. In both tables, the results are not much different from those presented above (when *γ* is between −0.2 and −0.3), although they do differ slightly for the most extreme values of *γ* and *c*. This can be seen in ${\hat{\beta }}_{t}$ (Table 2) when *γ* = −0.70, and in ${\hat{\beta }}_{t}\;\text{and}\;{\hat{\beta }}_{t^{2}}$ (Table 3) when *c* = 2. Since the extreme values are less probable, the first selection bias function is likely sufficient.

While the partly conditional model provides a description of how mean cognitive function in survivors depends on time and covariates like sex and education, it does not explain why these dependences arise. They could arise from multiple causes: differing initial outcomes in different types of subject; changes in outcome within subjects over time; and, importantly, differing hazards of death in different types of subject. For example, an association between being a woman (respectively, being older) and a faster decrease over time in mean outcome given survival could be partly due to mortality being higher in women (older subjects) with good cognitive function than in men (younger subjects) with good cognitive function. Thus, the outcome and death processes are interlinked. No single estimand can fully describe both processes simultaneously. For this reason, to better understand why dependencies arise, it could be of interest to supplement the results from a partly conditional model with estimates from a model for the hazard of death, as we show in Web Appendix I. In brief, the estimates from the supplementary survival analysis of the HRS data indicate that we can likely rule out differing hazards of death as one of the reasons for these dependencies.

## 7 Discussion

We have described several semi-parametric methods (IPW, CMOR, and AIPW) to make partly conditional inference for non-ignorable missing data. As in RRS and VRR, our methods use a tilt function that relates the distribution of an outcome at visit *t* among those who were last observed at some time before *t* to those who were observed at visit *t*. Unlike RRS and VRR, we distinguish between death and other types of missingness, and make partly conditional inference. We have demonstrated the validity of the proposed methods in simulation studies, and illustrated our method using data from the HRS.

There are many options for the parametrization of the selection bias function $q_{t}\left({\bar{O}}_{t-1},Y_{t};\gamma \right).$ Some authors argue that it is useful to elicit expert’s opinion about plausible selection bias functions (Rotnitzky et al., 2001; Shardell et al., 2010). Scharfstein et al. (2003) and Scharfstein et al. (2014) propose to use a low-dimensional parametrization of the selection bias function. They argue that a low dimension offers a more meaningful way for experts to encode their beliefs about the missingness process than a higher dimension. That is, it is desirable to restrict attention to a simple class of functions, so that the selection bias function is easily interpretable. As described in Scharfstein et al. (2003), “the aim is not to find the truth about this function, but to report an analysis which reasonably reflects an expert’s beliefs about selection bias.” In our data analysis, we used $q_{t}\left({\bar{O}}_{t-1},Y_{t};\gamma \right)=\gamma Y_{t};$ this was also used by Shardell et al. (2010) and Scharfstein et al. (2014). We also used $q_{t}\left({\bar{O}}_{t-1},Y_{t};\gamma \right)=\gamma_{1}R_{t-1}Y_{1}+\gamma_{2}\left(1-R_{t-1}\right)Y_{t},$ but obtained similar results.

Once the parametrization of $q_{t}\left({\bar{O}}_{t-1},Y_{t};\gamma \right)$ has been chosen, it is important to choose a plausible range of values for the sensitivity parameter. For example, the values can be selected based on experience from another similar data set analysis. When this is not possible, it might be useful to elicit expert opinion. See White (2014) for a comprehensive overview of this. Scharfstein et al. (2014) advise to compare the estimated average outcome among those who have dropped out with the observed average outcome among those who have not for different choices of *γ*. This allows experts to assess the plausibility of these imputed outcomes, and hence judge the plausibility of the sensitivity parameter value. In our HRS data analysis, we considered two simple selection bias functions, so that the magnitude and sign of the sensitivity parameter(s) were easy to interpret.

Alternatively, one could perform a “tipping point” analysis to investigate what values of the sensitivity parameter substantially change the conclusions about the statistical significance of the parameters of interest. Liublinska and Rubin (2014), for example, graphically illustrate an “enhanced tipping point” analysis for binary outcomes in combination with imputation procedures for the missing data.

Finally, although the AIPW estimators are doubly robust, they can be inconsistent when the missingness and regression models are both misspecified. Recently Vermeulen and Vansteelandt (2015) described how to estimate the parameters of these two models in a way that minimises the squared asymptotic bias of the doubly robust estimator even when both models are misspecified. It may be possible to adapt this method for our AIPW estimators.

## Supplementary Material

Web Appendix referenced in Sections 1, 3, and 6 is available with this article at the *Biometrics* website on Wiley Online Library.

Supplementary information

## Figures and Tables

**Table 1:** Analysis of HRS data using IEE and LMM

	IEE			LMM		
Param.	Estimate	SE	p-value	Estimate	SE	p-value
Int	11.959	0.521	0.00	12.078	0.493	0.00
*t*	−0.050	0.135	0.71	−0.261	0.112	0.02
*t^2^*	−0.018	0.008	0.02	−0.041	0.006	0.00
Age	−0.312	0.025	0.00	−0.312	0.024	0.00
Sex	0.034	0.198	0.86	0.059	0.199	0.77
Edu	0.696	0.030	0.00	0.678	0.028	0.00
*t*·age	−0.011	0.008	0.14	−0.036	0.006	0.00
*t*·sex	0.006	0.049	0.90	−0.069	0.042	0.09
*t*·edu	−0.014	0.007	0.05	0.003	0.006	0.56

**Table 2:** Simulation results for the monotone study with *n*=500 and true parameters *β*_1_ = 4.1843, *β*_2_ = −0.0877, *β*_3_ = −0.4225, *β*_4_ = −0.7836, *β*_5_ = −1.1552. Bias and empirical standard error (SE) are multiplied by 100. CP denotes coverage probability.

Param.	Misspecified models	IPW			CMOR			AIPW		
		
		Bias	SE	CP	Bias	SE	CP	Bias	SE	CP
*β*_2_	None	0.15	7.57	95.2	−0.04	7.42	95.6	0.15	7.57	95.2
	Missingness	6.49	7.59	86.1	−0.04	7.42	95.6	0.03	7.47	95.7
	Regression	0.15	7.57	95.2	8.05	7.46	82.8	0.32	7.62	95.3
	All	6.49	7.59	86.1	8.05	7.46	82.8	6.49	7.59	86.1
*β*_3_	None	0.83	11.27	90.9	−0.24	8.98	94.5	−0.03	9.55	93.1
	Missingness	10.30	9.37	79.0	−0.24	8.98	94.5	−0.12	9.11	94.1
	Regression	0.83	11.27	90.9	12.39	8.76	71.4	0.86	10.54	92.6
	All	10.30	9.37	79.0	12.39	8.76	71.4	10.09	9.04	79.6
*β*_4_	None	1.46	14.67	89.8	−0.55	10.72	95.3	−0.17	11.86	93.8
	Missingness	11.90	10.91	77.4	−0.55	10.72	95.3	−0.53	10.99	94.8
	Regression	1.46	14.67	89.8	14.39	10.24	69.8	1.43	13.85	92.5
	All	11.90	10.91	77.4	14.39	10.24	69.8	11.57	10.66	78.4
*β*_5_	None	2.59	16.63	90.5	−0.35	11.95	93.6	−0.03	13.85	94.3
	Missingness	13.30	12.25	78.6	−0.35	11.95	93.6	−0.29	12.31	95.5
	Regression	2.59	16.63	90.5	16.12	11.15	67.8	2.69	15.82	93.0
	All	13.30	12.25	78.6	16.12	11.15	67.8	12.97	11.90	80.6

*Note*: For *β*_1_: (bias×100, SE×100, CP) = (0.40, 5.91, 95.0) in all methods.

**Table 3:** Simulation results for the non-monotone study with *n*=500 and true parameters *β*_4_ = −1.2353, *β*_5_ = −1.8086. Bias and empirical standard error (SE) are multiplied by 100. CP denotes coverage probability. $\left\{m_{4}({\bar{O}}_{3}),\;h_{5}({\bar{O}}_{4})\right\}$ represents misspecification in the outcome regression model at visit 4 and misspecification in the missingness model at visit 5.

Param.	Misspecified models	IPW			CMOR			AIPW		
		
		Bias	SE	CP	Bias	SE	CP	Bias	SE	CP
*β*_4_	Neither	0.79	11.98	92.6	0.66	11.48	94.6	0.72	11.86	93.1
	$\left\{m_{4}({\bar{O}}_{3}),\;h_{5}({\bar{O}}_{4})\right\}$	0.79	11.98	92.6	13.28	11.09	73.9	0.87	11.87	92.8
	All	13.79	11.05	75.9	13.28	11.09	73.9	13.74	11.06	74.2
*β*_5_	Neither	1.35	14.00	91.6	0.95	13.37	93.3	1.11	13.62	92.2
	$\left\{m_{4}({\bar{O}}_{3}),\;h_{5}({\bar{O}}_{4})\right\}$	12.83	12.67	81.2	0.95	13.37	93.3	0.95	13.38	93.3
	All	12.83	12.67	81.2	12.15	12.79	79.1	12.77	12.67	79.5

**Table 4:** Sensitivity analysis for the non-monotone study with *n*=500 and true parameters *β*_1_ = 4.1843, *β*_2_ = −0.0877, *β*_3_ = −0.6753, *β*_4_ = −1.2353, *β*_5_ = −1.8086. Bias and empirical standard error (SE) are multiplied by 100. CP denotes coverage probability.

	IPW			CMOR			AIPW		
Parameter	Bias	SE	CP	Bias	SE	CP	Bias	SE	CP
*γ* = 0									
*β*_2_	7.31	8.29	85.9	7.24	8.09	85.6	7.31	8.29	85.9
*β*_3_	7.96	10.04	84.5	8.12	9.44	86.0	7.76	10.05	85.5
*β*_4_	8.90	11.68	86.2	8.71	11.15	86.4	8.43	11.58	86.1
*β*_5_	10.53	13.78	83.7	9.39	13.16	85.9	9.21	13.42	85.4
*γ* = −0.1									
*β*_2_	4.21	8.28	92.3	4.02	8.09	93.2	4.21	8.28	92.3
*β*_3_	4.28	10.10	91.2	4.33	9.50	92.7	4.15	10.10	91.2
*β*_4_	4.83	11.80	90.2	4.67	11.28	91.6	4.57	11.70	91.3
*β*_5_	5.93	13.86	88.8	5.15	13.23	91.5	5.15	13.49	90.0
*γ* = −0.3									
*β*_2_	−1.97	8.35	94.4	−2.36	8.20	94.4	−1.97	8.35	94.4
*β*_3_	−3.03	10.35	93.4	−3.17	9.82	94.1	−3.03	10.32	93.5
*β*_4_	−3.22	12.20	92.3	−3.31	11.74	93.7	−3.10	12.06	92.8
*β*_5_	−3.17	14.21	91.6	−3.21	13.59	93.6	−2.91	13.82	92.3
*γ* = −0.4									
*β*_2_	−5.05	8.44	89.9	−5.52	8.31	89.4	−5.05	8.44	89.9
*β*_3_	−6.66	10.53	89.4	−6.85	10.05	90.0	−6.57	10.48	89.0
*β*_4_	−7.20	12.47	89.0	−7.20	12.05	89.7	−6.88	12.31	88.9
*β*_5_	−7.64	14.49	88.0	−7.29	13.89	90.2	−6.89	14.08	90.2
*γ* = −0.5									
*β*_2_	−8.11	8.57	83.6	−8.65	8.46	83.7	−8.11	8.57	83.6
*β*_3_	−10.24	10.75	81.9	−10.45	10.32	83.1	−10.07	10.68	82.4
*β*_4_	−11.12	12.79	83.2	−11.01	12.41	84.5	−10.60	12.60	84.3
*β*_5_	−12.04	14.83	82.1	−11.29	14.25	84.1	−10.81	14.39	83.6

*Note:* For *β*_1_: (bias×100, SE×100, CP) = (0.01, 5.78, 94.5) in all methods (and *γ*).

**Table 5:** Parameter estimate (standard error) from model (equation (1)) for cognitive function fitted to HRS data

	Intercept	*t*	*t*^2^	Age	Sex	Edu	*t*·age	*t*·sex	*t*·edu
*γ*	IPW								

0.000	11.897 (0.458)	−0.125 (0.124)	−0.011 (0.010)	−0.302 (0.027)	0.029 (0.211)	0.701 (0.032)	−0.030 (0.009)	−0.101 (0.063)	−0.012 (0.007)
−0.050	11.787 (0.456)	−0.132 (0.120)	−0.014 (0.010)	−0.309 (0.027)	0.025 (0.213)	0.708 (0.032)	−0.028 (0.009)	−0.124 (0.062)	−0.012 (0.007)
−0.100	11.696 (0.457)	−0.151 (0.118)	−0.015 (0.010)	−0.315 (0.028)	0.021 (0.216)	0.712 (0.032)	−0.026 (0.009)	−0.145 (0.061)	−0.012 (0.007)
−0.150	11.618 (0.460)	−0.176 (0.119)	−0.016 (0.010)	−0.321 (0.028)	0.021 (0.220)	0.715 (0.033)	−0.023 (0.009)	−0.165 (0.061)	−0.011 (0.007)
−0.200	11.547 (0.464)	−0.200 (0.120)	−0.016 (0.010)	−0.325 (0.029)	0.026 (0.226)	0.715 (0.033)	−0.021 (0.010)	−0.181 (0.062)	−0.011 (0.008)
−0.250	11.484 (0.471)	−0.223 (0.123)	−0.015 (0.010)	−0.328 (0.029)	0.042 (0.234)	0.713 (0.034)	−0.019 (0.010)	−0.195 (0.063)	−0.010 (0.008)
−0.300	11.431 (0.479)	−0.245 (0.126)	−0.014 (0.010)	−0.329 (0.030)	0.067 (0.242)	0.710 (0.035)	−0.018 (0.010)	−0.206 (0.065)	−0.010 (0.008)

	CMOR								

0.000	12.057 (0.435)	−0.227 (0.115)	−0.006 (0.012)	−0.321 (0.025)	0.022 (0.199)	0.696 (0.031)	−0.013 (0.007)	−0.037 (0.048)	−0.014 (0.007)
−0.050	11.908 (0.439)	−0.229 (0.116)	−0.011 (0.011)	−0.324 (0.026)	0.007 (0.202)	0.705 (0.031)	−0.016 (0.007)	−0.053 (0.049)	−0.013 (0.007)
−0.100	11.771 (0.444)	−0.232 (0.118)	−0.014 (0.011)	−0.326 (0.026)	−0.009 (0.207)	0.713 (0.032)	−0.017 (0.008)	−0.067 (0.051)	−0.013 (0.007)
−0.150	11.640 (0.449)	−0.233 (0.121)	−0.015 (0.011)	−0.326 (0.026)	−0.021 (0.212)	0.718 (0.032)	−0.018 (0.008)	−0.079 (0.053)	−0.014 (0.007)
−0.200	11.510 (0.453)	−0.230 (0.123)	−0.017 (0.011)	−0.326 (0.027)	−0.026 (0.217)	0.721 (0.032)	−0.019 (0.008)	−0.089 (0.055)	−0.014 (0.008)
−0.250	11.377 (0.457)	−0.225 (0.126)	−0.017 (0.011)	−0.324 (0.027)	−0.025 (0.222)	0.724 (0.033)	−0.019 (0.008)	−0.096 (0.057)	−0.015 (0.008)
−0.300	11.251 (0.461)	−0.224 (0.130)	−0.017 (0.011)	−0.322 (0.027)	−0.023 (0.226)	0.725 (0.033)	−0.019 (0.008)	−0.100 (0.059)	−0.016 (0.008)

	AIPW								

0.000	11.851 (0.453)	−0.118 (0.125)	−0.010 (0.011)	−0.308 (0.026)	0.072 (0.209)	0.705 (0.032)	−0.026 (0.008)	−0.105 (0.061)	−0.014 (0.008)
−0.050	11.721 (0.449)	−0.130 (0.119)	−0.013 (0.010)	−0.314 (0.026)	0.064 (0.209)	0.713 (0.032)	−0.025 (0.008)	−0.123 (0.058)	−0.014 (0.008)
−0.100	11.608 (0.448)	−0.154 (0.116)	−0.015 (0.010)	−0.319 (0.027)	0.054 (0.211)	0.720 (0.032)	−0.022 (0.008)	−0.136 (0.056)	−0.013 (0.007)
−0.150	11.500 (0.450)	−0.177 (0.116)	−0.015 (0.010)	−0.323 (0.027)	0.043 (0.214)	0.725 (0.032)	−0.020 (0.008)	−0.145 (0.055)	−0.014 (0.007)
−0.200	11.391 (0.455)	−0.195 (0.118)	−0.015 (0.011)	−0.325 (0.027)	0.037 (0.218)	0.728 (0.032)	−0.019 (0.008)	−0.150 (0.055)	−0.014 (0.007)
−0.250	11.282 (0.461)	−0.204 (0.121)	−0.015 (0.011)	−0.325 (0.027)	0.035 (0.224)	0.730 (0.033)	−0.018(0.008)	−0.153 (0.056)	−0.015 (0.008)
−0.300	11.180 (0.468)	−0.208 (0.125)	−0.015 (0.011)	−0.324 (0.028)	0.041 (0.230)	0.731 (0.033)	−0.018 (0.008)	−0.157 (0.058)	−0.015 (0.008)
