# Supplementary material for: Semi-Parametric Methods of Handling Missing Data in Mortal Cohorts under Non-Ignorable Missingness
Source: Biometrics. Author manuscript; Available in PMC 2019 Apr 24. (PMC6481558; doi:10.1111/biom.12891)
Supplement: Supplementary information [file NIHMS82523-supplement-Supplementary_information.pdf]

Biometrics 000, 000–000 DOI: 000
000 0000

Web-based Supplementary Materials for "Semi-parametric methods of handling missing data in mortal cohorts under non-ignorable missingness"

Lan Wen

MRC Biostatistics Unit, University of Cambridge, IPH Forvie Site, Robinson Way, Cambridge CB2 0SR, UK

email: lan.wen@mrc-bsu.cam.ac.uk

and

Shaun R. Seaman

MRC Biostatistics Unit, University of Cambridge, IPH Forvie Site, Robinson Way, Cambridge CB2 0SR, UK

email: shaun.seaman@mrc-bsu.cam.ac.uk

Appendices

Appendix A

Details about the SACE

A.1 Example that compares the partly conditional mean and the SACE

Let Z be an indicator of exposure (or treatment) where $Z = 1$ if a subject is exposed and 0 otherwise, and let $Y(Z)$ and $A(Z)$ be the counterfactual outcome and vital status under Z. For example $A(1) = 1$ means that the subject would have been alive had he/she been exposed and $A(1) = 0$ means that the subject would have died had he/she been exposed. The four principal strata are:

S1. Always survivors: those who would survive regardless of exposure status ($A(0) = A(1) = 1$)

S2. Survivors only if $Z = 1$: those who would survive only if exposed ($A(0) = 0, A(1) = 1$)

S3. Survivors only if $Z = 0$: those who would survive only if unexposed ($A(0) = 1, A(1) = 0$)

S4. Always diers: those who would die regardless of exposure status ($A(0) = A(1) = 0$)

Suppose that higher outcome values are more beneficial than low values, and assume that an exposure never harms survival (i.e. number of subjects in S3 is 0). Further suppose that the individuals in S1 have high (better) outcomes if exposed and moderate outcomes if not; and the individuals in S2 have low outcomes if exposed. A similar setting is found in Egleston *and others* (2009).

Table 1 shows a hypothetical observational study of the scenario described above, where half of the people in the study are exposed and the other half is unexposed. Suppose that Y is an outcome of interest (e.g. health status) and assume that undefined post-death outcomes are represented by $*$.

[Table 1 about here.]

The difference between the partly conditional means in the exposed and the unexposed is

$E(Y \mid Z=1, A=1) - E(Y \mid Z=0, A=1) = 0$, whereas the SACE is $E\{Y(1) - Y(0) \mid S1\} = 3$. The appropriate choice of estimand analysis method depend on the research aim (Kurland *and others*, 2009).

If Z is a treatment indicator, then it is obvious that treatment ($Z = 1$) is better than placebo ($Z = 0$) because the treatment has a positive effect on the outcomes in the always survivors and on survival, and hence the partly conditional mean would be misleading because it implies that the effect of the treatment on the outcome is not different from the placebo. In this case the SACE would be more appropriate than the partly conditional mean.

Now suppose that Z is an indicator of education level (i.e. $Z = 1$ if subjects have a high level of education and 0 otherwise). In reality, Z would include many other variables such as sex and age, but for the purpose of illustrating the difference between the estimand, we consider one variable – education. Suppose that policy-makers would like to know how the average outcomes in the oldest-old differ between those with high and low education levels so that they know approximately how much resources to allocate to surviving subjects with high/low levels of education. Higher (better) outcome would imply lower requirement for resources. Then the SACE is misleading as a measure because it suggests that people with more education would require less health support than people with less education. A policy derived from the SACE would neglect those subjects with a high level of education and low outcome ($Y = 2$). In this case the partly conditional mean would provide a more fairer comparison.

A.2 The SACE: methods and assumptions

Methods to estimate the SACE either (1) calculate bounds on the SACE (Zhang and Rubin, 2003; Chiba, 2012; Yang and Small, 2016), (2) make assumptions that allow the SACE to be identifiable and estimable (Mattei and Mealli, 2007; Zhang *and others*, 2009; Ding *and*

others, 2011), or (3) conduct sensitivity analysis around assumptions about the distribution of the outcome or survival process (Egleston *and others*, 2007; Jemiai *and others*, 2007; Chiba and VanderWeele, 2011). The SACE is not identified without untestable assumptions, e.g. the stable unit treatment value assumption (SUTVA) and the no unmeasured confounders assumption. SUTVA says that a subject's potential outcomes do not depend on the exposure status of other subjects and that there are no multiple versions of treatment; no unmeasured confounders says that given observed covariates, exposure is independent of potential outcomes. With the exception of Zhang *and others* (2009) who make parametric modelling assumptions, the aforementioned papers assume monotonicity. Monotonicity says that all subjects who would survive if exposed would survive if unexposed. Zhang and Rubin (2003), Chiba (2012) and Yang and Small (2016) also make the ranked average scores assumption, which says that the average potential outcome when exposed is better in the S1 group (always survivors) than in the S2 group, and that the average potential outcome when not exposed is better for the S1 group than for the S3 group. A table summarizing methods to estimate the SACE can be found below.

It would be interesting to estimate the SACE for education in the HRS. However, while we would not exclude the possibility of doing this, there would be difficulties. First, there is the problem of multiple versions of treatment. That is, it is unclear what intervention on education one would be considering. Different interventions could be expected to produce different causal effects on cognitive function. For example, allow access to high-quality preparatory schools for everyone, encouraging women and people of colour to attend more school, or granting subsidized education might well have different causal effects on cognitive function in later life. If an estimate of SACE were to be obtained from the HRS data, it is unclear to which (if any) of these possible interventions it would relate. Second, as outlined above, quite strong assumptions are needed to estimate a SACE (e.g. no unmeasured

confounders) and one might be a little wary about making such assumptions to estimate a SACE for education from the HRS data. The aim in our paper is arguably more modest: to *describe* associations between outcomes and covariates.

A.3 Table of methods and assumptions for identifying the SACE

In the following table, X is vector of auxiliary variables, Z is exposure status ($Z = 1$ if exposed, 0 otherwise), $Y(Z)$ is potential outcome under exposure Z, $A(Z)$ is potential vital status under exposure Z ($A(Z) = 1$ if a subject would be alive had he/she been given exposure Z). All of the methods described in this section assume SUTVA and randomization (or no unmeasured confounders).

An estimand related to the SACE is the complier average causal effect (CACE), which is relevant when subjects in a randomized trial fail to adhere to their treatment assignment. Let Z be a binary treatment assignment indicator ($Z = 1$ if assigned to active treatment, $Z = 0$ if assigned to control treatment) and $A(Z)$ be the potential treatment compliance indicator. That is, $A(1) = 1$ if the subject would take the active treatment if assigned to active treatment ($A(1) = 0$ if s/he would instead take the control treatment), and $A(0) = 1$ if the subject would take the control treatment if assigned to control treatment ($A(0) = 0$ if s/he would instead take the active treatment).

There are four principal strata that arise from compliance/non-compliance:

(1) Compliers: those who receive the treatment s/he is assigned ($A(0) = A(1) = 1$)
(2) Always-takers: those who receive active treatment, regardless of assignment (A(0)=0,A(1)=1)
(3) Never-takers: those who receive control treatment, regardless of assignment (A(0)=1,A(1)=0)
(4) Defiers: those who receive the opposite treatment s/he is assigned ($A(0) = A(1) = 0$)

CACE is then the effect of the treatment on an outcome Y in the compliers. There is an immediate parallel between SACE and CACE in that survival is analogous to compliance and death is analogous to non-compliance. However, unlike for death, both potential outcomes

are defined for subjects in all four strata. Many of the methods in the following table are described in the context of the CACE, but for the purpose of this paper we will describe them in the context of the SACE.

Paper	Assumptions	Method
Zhang and Rubin (2003)	-Monotonicity<br>-Ranked average score	-Identifying bounds of SACE<br>-Randomized trial
Imai (2008)	-Monotonicity<br>-Stochastic dominance (similar to, but weaker than, ranked score average)	-Identifying bounds for quantiles of the causal effect in always survivors<br>-Randomized trial
Chiba (2012)	-Monotonicity<br>-Ranked average score<br>-Assumptions about stratum sizes (to narrow bounds)	-Identifying bounds of SACE<br>-Randomized trial
Yang and Small (2016)	-Monotonicity<br>-Ranked average score assumptions for two time points	-Identifying bounds of SACE<br>-Survival data before and after measurements of outcome used to narrow bounds<br>-Observational study
Mattei and Mealli (2007)	-Monotonicity<br>-Latent ignorability: within PS missingness is ignorable<br>-Stochastic dominance<br>-Other assumptions about compliers and non-compliers for those who are given active treatment	-Make assumptions so SACE is identified<br>-Bayesian approach to draw SACE<br>-Complications from death and from non-compliance<br>-Randomized trial
Zhang *and others* (2009)	-P(belonging to a PS $\mid X$) obey multinomial logit model<br>-$Y(1), Y(0)$ in S1 are bivariate normal with mean linear in X<br>-$Y(1)$ in S2, $Y(0)$ in S3 are normally dist. with mean linear in X	-Make assumptions so SACE is identified<br>-Parametric model assumptions to draw SACE<br>-Randomized trial
Ding *and others* (2011)	-Monotonicity (relaxed if (i) and/or (ii) hold and X is cts.)<br>(i) $X \perp\!\!\!\perp Y \mid$ PS and Z<br>(ii) Distribution of X in S1 and in S2 are different	-Make assumptions so SACE is identified<br>-Randomized trial<br>-SACE identified if (i), (ii) hold
Hayden *and others* (2005)	-Explainable nonrandom survival:<br>(i) $A(1) \perp\!\!\!\perp A(0) \mid X$<br>(ii) $A(Z) \perp\!\!\!\perp Y(1-Z) \mid X, \{A(1-Z)=1\}$, for	-Sensitivity analysis around dist. of Y<br>-ML for joint dist. of $Y(1), Y(0), A(1), A(0)$<br>-Randomized trial
Egleston *and others* (2007)	-Monotonicity<br>-$E(Y(1)\|S2)\tau = E(Y(1)\|S1)$, e.g. $\tau = 1$<br>-Outcomes are MAR	-Sensitivity analysis around dist. of Y<br>-τ is sensitivity parameter<br>-Observational study
Egleston *and others* (2009)	-Monotonicity<br>-$E(Y(1)\|S2, X)\tau = E(Y(1)\|S1, X)$, e.g. $\tau = 1$<br>-Outcome Y is MAR<br>-Missing confounder may be MNAR	-Sensitivity analysis around dist. of Y<br>-Sensitivity analysis around MAR assumption for missing confounder<br>-Observational study
Chiba and VanderWeele (2011)	-Monotonicity<br>-$E(Y\|Z=1, A=1) = E(Y\|Z=0, A=1) - \alpha$<br>where $\alpha = E(Y(1)\|Z=1, A(1)=1) - E(Y(1)\|Z=0, S1)$	-Sensitivity analysis around dist. of Y<br>-α is sensitivity parameter<br>-Randomized trial<br>-Observational study
Gilbert *and others* (2003)	-Monotonicity<br>-logit $P(A(1)\|Y(0)=y, A(0)=1) = \alpha + \beta y$	-Sensitivity analysis around survival dist.<br>-α is estimated, β is sensitivity parameter<br>-Randomized trial
Jemiai *and others* (2007)	-Monotonicity<br>-logit $P(A(1)\|Y(0)=y, A(0)=1, X=x) = f(X;\alpha) + g(y, x; \phi)$<br>-SACE can be a function of X	-Sensitivity analysis around survival dist.<br>-Generalization of Gilbert *and others* (2003) to allow for baseline covariates<br>-α is estimated; ϕ is sensitivity param.<br>-Randomized trial
Shepherd *and others* (2008)	-logit$(P(A(1)=1\|Y(0)=y, A(0)=1, X)) = \alpha_1 + \beta_1 y$ and<br>-logit$(P(A(0)=1\|Y(0)=y, A(0)=1, X)) = \alpha_2 + \beta_2 y$<br>-$P(A(0)=1\|A(1)=1) = \phi$	-Sensitivity analysis around survival dist.<br>-β_1, β_2, ϕ are sensitivity param.<br>-α_1, α_2 are estimated<br>-Randomized trial

PS: Principal strata (or stratum)
ML: Maximum likelihood

Appendix B

Asymptotic distribution of IPW estimators

Let U^{IPW} equal to estimating equations (4). Under standard regularity conditions,

$$\sqrt{n}(\hat{\beta}-\beta_0) \xrightarrow{d} MVN\left\{0, \Sigma^{-1}_{U^{IPW}_{\beta}}\Omega_{U^{IPW}}(\Sigma^{-1}_{U^{IPW}_{\beta}})^T\right\}$$

where

$$\Sigma_{U^{IPW}_{\beta}} = E\left(\frac{\partial U^{IPW}(\alpha_0,\beta_0)}{\partial\beta}\right), \qquad A_{U^{IPW}_{\alpha}} = E\left(\frac{\partial U^{IPW}(\alpha_0,\beta_0)}{\partial\alpha}\right), \qquad B_Q = E\left(\frac{\partial Q(\alpha_0)}{\partial\alpha}\right)$$

$$\Omega_{U^{IPW}} = E\left[\left\{U^{IPW}(\alpha_0,\beta_0) - A_{U^{IPW}_{\alpha}}B_Q^{-1}Q(\alpha_0)\right\}^{\otimes 2}\right]$$

and α_0 and β_0 are the true values of α and β.

Proof. Under suitable regularity conditions, expand equation (4) around the true value β_0 (fixing $\hat{\alpha}$, by mean value expansion):

$$0 = \sum_{i=1}^n U_i^{IPW}(\hat{\alpha},\hat{\beta}) = \sum_{i=1}^n U_i^{IPW}(\hat{\alpha},\beta_0) + \sum_{i=1}^n \frac{\partial U_i^{IPW}(\hat{\alpha},\tilde{\beta})}{\partial\beta}(\hat{\beta}-\beta_0)$$

$$\therefore \sqrt{n}(\hat{\beta}-\beta_0) = -\left\{\frac{1}{n}\sum_{i=1}^n \frac{\partial U_i^{IPW}(\hat{\alpha},\tilde{\beta})}{\partial\beta}\right\}^{-1}\left\{\frac{1}{\sqrt{n}}\sum_{i=1}^n U_i^{IPW}(\hat{\alpha},\beta_0)\right\}$$

where $\tilde{\beta}$ is an intermediate value between β_0 and $\hat{\beta}$. Next, we expand around α_0.

$$\sum_{i=1}^n U_i^{IPW}(\hat{\alpha},\beta_0) = \sum_{i=1}^n U_i^{IPW}(\alpha_0,\beta_0) + \sum_{i=1}^n \frac{\partial U_i^{IPW}(\tilde{\alpha},\beta_0)}{\partial\alpha}(\hat{\alpha}-\alpha_0)$$

$$\therefore \sqrt{n}(\hat{\beta}-\beta_0) = -\left\{n^{-1}\sum_{i=1}^n \frac{\partial U_i^{IPW}(\hat{\alpha},\tilde{\beta})}{\partial\beta}\right\}^{-1}\left\{\frac{1}{\sqrt{n}}\sum_{i=1}^n U_i^{IPW}(\alpha_0,\beta_0) + n^{-1}\sum_{i=1}^n \frac{\partial U_i^{IPW}(\tilde{\alpha},\beta_0)}{\partial\alpha}\sqrt{n}(\hat{\alpha}-\alpha_0)\right\} \quad \text{(B.1)}$$

We can also expand equation (3.1) around α_0 to get:

$$\sqrt{n}(\hat{\alpha}-\alpha_0) = -\left\{\frac{1}{n}\sum_{i=1}^n \frac{\partial Q_i(\tilde{\alpha})}{\partial\alpha}\right\}^{-1}\left\{\frac{1}{\sqrt{n}}\sum_{i=1}^n Q_i(\alpha_0)\right\}$$

where $\tilde{\alpha}$ is an intermediate value between α_0 and $\hat{\alpha}$. Substituting this into equation (B.1)

and using law of large of numbers, we get

$$
\begin{aligned}
&\sqrt{n}(\hat{\beta}-\beta_0)\\
&= -\left(\frac{1}{n}\sum_{i=1}^{n}\frac{\partial U_i^{IPW}(\hat{\alpha},\tilde{\beta})}{\partial\beta}\right)^{-1}\left[\frac{1}{\sqrt{n}}\sum_{i=1}^{n}U_i^{IPW}(\alpha_0,\beta_0)-\frac{1}{n}\sum_{i=1}^{n}\frac{\partial U_i^{IPW}(\tilde{\alpha},\beta_0)}{\partial\alpha}\left\{\left(\frac{1}{n}\sum_{i=1}^{n}\frac{\partial Q_i(\tilde{\alpha})}{\partial\alpha}\right)^{-1}\left(\frac{1}{\sqrt{n}}\sum_{i=1}^{n}Q_i(\alpha_0)\right)\right\}\right]\\
&= -E\left(\frac{\partial U^{IPW}(\alpha_0,\beta_0)}{\partial\beta}\right)^{-1}\left[\frac{1}{\sqrt{n}}\sum_{i=1}^{n}U_i^{IPW}(\alpha_0,\beta_0)-E\left(\frac{\partial U^{IPW}(\alpha_0,\beta_0)}{\partial\alpha}\right)\left\{E\left(\frac{\partial Q(\alpha_0)}{\partial\alpha}\right)^{-1}\left(\frac{1}{\sqrt{n}}\sum_{i=1}^{n}Q_i(\alpha_0)\right)\right\}\right]+o_p(1)\\
&= n^{-\frac{1}{2}}\sum_{i=1}^{n}\left[-E\left(\frac{\partial U^{IPW}(\alpha_0,\beta_0)}{\partial\beta}\right)^{-1}\left\{U_i^{IPW}(\alpha_0,\beta_0)-E\left(\frac{\partial U^{IPW}(\alpha_0,\beta_0)}{\partial\alpha}\right)E\left(\frac{\partial Q(\alpha_0)}{\partial\alpha}\right)^{-1}Q_i(\alpha_0)\right\}\right]+o_p(1)
\end{aligned}
$$

Since $E\{U(\alpha_0,\beta_0)\}=0$ and $E\{U(\alpha_0)\}=0$, we can deduce that the influence function $\hat{\beta}$ is

$$
-E\left(\frac{\partial U^{IPW}(\alpha_0,\beta_0)}{\partial\beta}\right)^{-1}\left\{U^{IPW}(\alpha_0,\beta_0)-E\left(\frac{\partial U^{IPW}(\alpha_0,\beta_0)}{\partial\alpha}\right)E\left(\frac{\partial Q(\alpha_0)}{\partial\alpha}\right)^{-1}Q(\alpha_0)\right\} \tag{B.2}
$$

and the asymptotic distribution of $\hat{\beta}$ follows.

Note: If we had estimated α using maximum likelihood, the content in $\{\cdot\}$ of equation (B.2) would be equal the projection of $U(\alpha,\beta)$ onto the linear subspace spanned by elements of the score function, Λ_α. Then $\{\cdot\}$ of equation (B.2) simplifies to

$$
U^{IPW}(\alpha_0,\beta_0)-E\{U^{IPW}(\alpha_0,\beta_0)S^T(\alpha_0)\}E\{S(\alpha_0)S^T(\alpha_0)\}^{-1}S(\alpha_0)
$$

where $S(\alpha_0)$ is made up of the score function of the probability density function of missingness process. By the Multivariate Pythagorean Theorem (Tsiatis, 2006), variance matrix of $\{\cdot\}$ is positive semi-definite and therefore ${\Sigma_{U_\beta^{IPW}}}^{-1}E\left\{U^{IPW}(\alpha_0,\beta_0)^{\otimes 2}\right\}\left({\Sigma_{U_\beta^{IPW}}}^{-1}\right)^T$, where $\Sigma_{U_\beta^{IPW}}=E\left(\dfrac{\partial U^{IPW}(\alpha_0,\beta_0)}{\partial\beta}\right)^{-1}$, would overestimate the asymptotic variance.

Appendix C

Details about Conditional Mean Outcome Regression

Equation (3) implies the following lemma, which relates the expected outcome at visit t given history $\bar{O}_{t-1}$ in survivors who drop out just after visit $t-1$ to the expected outcome in survivors who are observed at visit t.

Lemma 1: *If Assumption 1 holds, then*

$$E(Y_t \mid \bar{O}_{t-1}, R_{t-1} = 1, R_t = 0, A_t = 1) = \frac{E[Y_t \exp\{q_t(\bar{O}_{t-1}, Y_t)\} \mid \bar{O}_{t-1}, R_t = 1, A_t = 1]}{E[\exp\{q_t(\bar{O}_{t-1}, Y_t)\} \mid \bar{O}_{t-1}, R_t = 1, A_t = 1]}$$

If $q_t(\bar{O}_{t-1}, Y_t; \gamma) = 0$, then conditional on $\bar{O}_{t-1}$ and survival at visit t, subjects who drop out between visits $t-1$ and t have the same Y_t distribution as those who are observed at visit t. If $q_t(\bar{O}_{t-1}, Y_t; \gamma)$ is an increasing (decreasing) function of Y_t, then this indicates that subjects who drop out between visits $t-1$ and t tend to have larger (smaller) values of Y_t than those who are observed at visit t.

Let $m_t(\bar{O}_s; \theta_{t,s})$ $(0 \leqslant s < t \leqslant J)$ be a model for $m_t(\bar{O}_s) = E(Y_t \mid \bar{O}_s, R_s = 1, R_{s+1} = 0, A_t = 1)$ with unknown parameter $\theta_{t,s}$. Lemma 1 implies that $\theta_{t,t-1}$ $(t = 1, 2, \ldots, J)$ can be estimated by regressing Y_t on $\bar{O}_{t-1}$ in the subset of individuals with $R_t = 1$, weighting each of these individuals by $\exp\{q_t(\bar{O}_{t-1}, Y_t)\}$. Let $\hat{\theta}_{t,t-1}$ denote the resulting estimate of $\theta_{t,t-1}$.

The following lemma relates the expected outcome at visit t given history $\bar{O}_s$ $(0 \leqslant s \leqslant t-2$ and $t \leqslant J)$ in survivors who drop out just after visit s to the expectation in those who are observed at visit $s+1$.

Lemma 2: *If Assumption 1 and mortal-cohort NFD hold, then for* $s \leqslant t-2$,

$$E(Y_t \mid \bar{O}_s, R_s = 1, R_{s+1} = 0, A_t = 1)$$
$$= \frac{E_{Y_{s+1}}\left[E(Y_t \mid \bar{O}_s, Y_{s+1}, R_{s+1} = 1, A_t = 1) \exp\{q_{s+1}(\bar{O}_s, Y_{s+1})\} \mid \bar{O}_s, R_{s+1} = 1, A_t = 1\right]}{E[\exp\{q_{s+1}(\bar{O}_s, Y_{s+1})\} \mid \bar{O}_s, R_{s+1} = 1, A_t = 1]}$$

Setting $s = t-2$, Lemma 2 indicates that, if $m_t(\bar{O}_{t-1})$ were known, then $\theta_{t,t-2}$ $(t = 2, \ldots, J)$ could be estimated by weighted regression after first imputing the missing Y_t value in each

subject with $R_{t-1} = 1$, $R_t = 0$ and $A_t = 1$ as its corresponding expectation $m_t(\bar{O}_{t-1})$. Specifically, Y_t (or, if missing, its imputed value, $m_t(\bar{O}_{t-1})$) would be regressed on $\bar{O}_{t-2}$ in the subset of individuals with $R_{t-1} = 1$ and $A_t = 1$, weighting each individual by $\exp\{q_{t-1}(\bar{O}_{t-2}, Y_{t-1})\}$. In practice, $m_t(\bar{O}_{t-1})$ is unknown. However, if $\hat{\theta}_{t,t-1}$ has previously been calculated using the weighted regression method described above (following Lemma 1), then $m_t(\bar{O}_{t-1})$ can be replaced by $m_t(\bar{O}_{t-1}; \hat{\theta}_{t,t-1})$.

A similar procedure can then be used to estimate $\theta_{t,t-3}$, then $\theta_{t,t-4}$, and so on. In detail, the CMOR procedure is as follows.

(1) For each $t = 1, \ldots, J$, let $Y_t^* = Y_t$ if $R_t = 1$ and let Y_t^* be missing otherwise. Set $a = 1$.

(2) For each $t = 1, \ldots, J$ in turn, let $s = t - a$ and calculate $\hat{\theta}_{t,s}$ by regressing Y_t^* on $\bar{O}_s$ in those subjects with $R_s = A_t = 1$ using weights $\exp\{q_{s+1}(\bar{O}_s, Y_{s+1}^*)\}$. If Y_t^* is still missing and $R_s = A_t = 1$, set $Y_t^* = m_t(\bar{O}_s; \hat{\theta}_{t,s})$.

(3) If $a < J$, let $a = a + 1$ and return to step 2.

Note that this procedure does not impute post-death outcomes (i.e. Y_t^* is still missing when $A_t = 0$). The following Lemma summarizes the procedure described above.

Lemma 3: *If mortal-cohort NFD holds, the selection bias function and the sensitivity parameter γ are correctly chosen, and the regression models are correctly specified, then the estimator $\hat{\theta}_{t,t-1}$ that solves*

$$\sum_{i=1}^{n} M_{it}\left(\theta_{t,t-1}\right) = \sum_{i=1}^{n} A_{it} R_{it} \exp\{q_t(\bar{O}_{i,t-1}, Y_{it}; \gamma)\} \left\{Y_{it} - m_t\left(\bar{O}_{i,t-1}; \theta_{t,t-1}\right)\right\} d_t(\bar{O}_{i,t-1}) = 0$$

and subsequently for $s < t - 1$, the estimator $\hat{\theta}_{t,s}$ that solves

$$\sum_{i=1}^{n} M_{it}\left(\theta_{t,s}\right) = \sum_{i=1}^{n} A_{it} R_{i,s+1} \exp\{q_{s+1}(\bar{O}_{is}, Y_{i,s+1}; \gamma)\} \times \left\{R_{it} Y_{it} + \sum_{l=s+1}^{t-1} R_{il}(1 - R_{i,l+1}) m_t\left(\bar{O}_{il}; \hat{\theta}_{t,l}\right) - m_t\left(\bar{O}_{is}; \theta_{t,s}\right)\right\} d_t(\bar{O}_{is}) = 0$$

where $d_t(\bar{O}_s)$ – a function of $\bar{O}_s$ – has the same dimension as $\theta_{t,s}$ ($\forall s < t$), will be consistent.

After imputing the missing outcomes as described above, the parameter β in the model of interest can be estimated by applying IEE to the imputed data set with Y_{it} replaced with $Y^*_{it} = R_{it}Y_{it} + \sum_{s=1}^{t-1} R_{is}(1 - R_{i,s+1})m_t(\bar{O}_{is}; \hat{\theta}_{t,s})$. That is, by solving the estimating equations

$$\sum_{i=1}^{n}\sum_{t=1}^{J} A_{it}\frac{\partial \mu_{it}}{\partial \beta}(Y^*_{it} - \mu_{it}) = 0 \tag{C.1}$$

Theorem 1: *If mortal-cohort NFD holds, the selection bias function and the sensitivity parameter γ are correctly chosen, and the regression models are correctly specified, then the estimator $\hat{\beta}$ defined as the solution to estimating equations (C.1) will be consistent.*

C.1 Asymptotic distribution of CMOR estimator

Let U^{CMOR} equal to estimating equations (C.1). Under standard regularity conditions,

$$\sqrt{n}(\hat{\beta} - \beta_0) \longrightarrow MVN\left\{0, \Sigma^{-1}_{U^{CMOR}_{\beta}}\Omega_{U^{CMOR}}(\Sigma^{-1}_{U^{CMOR}_{\beta}})^T\right\}$$

where $\Sigma_{U^{CMOR}} = E\left\{\frac{\partial U^{CMOR}(\theta_0,\beta_0)}{\partial \beta}\right\}$, $\quad A_{U^{CMOR}_{\theta}} = E\left\{\frac{\partial U^{CMOR}(\theta_0,\beta_0)}{\partial \theta}\right\}$, $\quad B_M = E\left\{\frac{\partial M(\theta_0)}{\partial \theta}\right\}$

$$\Omega_{U^{CMOR}} = E\left[\left\{U^{CMOR}(\theta_0, \beta_0) - A_{U^{CMOR}_{\theta}}B_M^{-1}M(\theta_0)\right\}^{\otimes 2}\right]$$

and θ_0 and β_0 are the true values of θ and β. Proof is analogous to that of the IPW estimator.

Appendix D

Proofs of Lemmas 1 and 2

Proof. **Lemma 1**

From Bayes' Theorem

$$\frac{P(R_t = 0|R_{t-1} = 1, Y_t, \bar{O}_{t-1}, A_t = 1)}{P(R_t = 1|R_{t-1} = 1, Y_t, \bar{O}_{t-1}, A_t = 1)} = \frac{f(Y_t|R_t = 0, \bar{O}_{t-1}, R_{t-1} = 1, A_t = 1)}{f(Y_t|R_t = 1, \bar{O}_{t-1}, A_t = 1)} \frac{P(R_t = 0|\bar{O}_{t-1}, R_{t-1} = 1, A_t = 1)}{P(R_t = 1|\bar{O}_{t-1}, R_{t-1} = 1, A_t = 1)}$$

Then it can be shown that

$$f(Y_t|R_t = 0, \bar{O}_{t-1}, R_{t-1} = 1, A_t = 1) = \frac{f(Y_t|R_t = 1, \bar{O}_{t-1}, A_t = 1) \exp\{q_t(\bar{O}_{t-1}, Y_t)\}}{E[\exp\{q_t(\bar{O}_{t-1}, Y_t)\}|\bar{O}_{t-1}, R_t = 1, A_t = 1]}$$

We then multiply the left- and right-hand side by Y_t, then integrate both sides with respect to Y_t to obtain Lemma 1.

Proof. **Lemma 2**

From Bayes' Theorem

$$\frac{P(R_{s+1} = 0|R_s = 1, Y_{s+1}, \ldots, Y_t, \bar{O}_s, A_t = 1)}{P(R_{s+1} = 1|R_s = 1, Y_{s+1}, \ldots, Y_t, \bar{O}_s, A_t = 1)} = \frac{f(Y_{s+1}, \ldots, Y_t|R_{s+1} = 0, \bar{O}_s, R_s = 1, A_t = 1)}{f(Y_{s+1}, \ldots, Y_t|R_{s+1} = 1, \bar{O}_s, A_t = 1)} \frac{P(R_{s+1} = 0|\bar{O}_s, R_s = 1, A_t = 1)}{P(R_{s+1} = 1|\bar{O}_s, R_s = 1, A_t = 1)}$$

This implies that

$$\frac{P(R_{s+1} = 0|R_s = 1, Y_{s+1}, \bar{O}_s, A_{s+1} = 1)}{P(R_{s+1} = 1|R_s = 1, Y_{s+1}, \bar{O}_s, A_{s+1} = 1)} = \frac{f(Y_{s+1}, \ldots, Y_t|R_{s+1} = 0, \bar{O}_s, R_s = 1, A_t = 1)}{f(Y_{s+1}, \ldots, Y_t|R_{s+1} = 1, \bar{O}_s, A_t = 1)} \frac{P(R_{s+1} = 0|\bar{O}_s, R_s = 1, A_t = 1)}{P(R_{s+1} = 1|\bar{O}_s, R_s = 1, A_t = 1)}$$

by mortal cohort NFD. Then it can be shown that

$$f(Y_{s+1}, Y_t|R_{s+1} = 0, \bar{O}_s, R_s = 1, A_t = 1) = \frac{f(Y_{s+1}, Y_t|R_{s+1} = 1, \bar{O}_s, A_t = 1) \exp\{q_{s+1}(\bar{O}_s, Y_{s+1})\}}{E[\exp\{q_{s+1}(\bar{O}_s, Y_{s+1})\}|\bar{O}_s, R_{s+1} = 1, A_t = 1]}$$

Integrate with respect to Y_{s+1}, we obtain

$$f(Y_t|R_{s+1} = 0, \bar{O}_s, R_s = 1, A_t = 1) = \frac{\int f(Y_{s+1}, Y_t|R_{s+1} = 1, \bar{O}_s, A_t = 1) \exp\{q_{s+1}(\bar{O}_s, Y_{s+1})\} dY_{s+1}}{E[\exp\{q_{s+1}(\bar{O}_s, Y_{s+1})\}|\bar{O}_s, R_{s+1} = 1, A_t = 1]}$$

Then multiplying both sides by Y_t and integrating with respect to Y_t, we obtain Lemma 2

Appendix E

Unbiasedness of AIPW estimating equations for monotone missing data

For simplicity, we assume that $R_1 = 1$ and that $\sum_{t=1}^{0}\{\cdot\} = 1$, but note that this proof can be easily adapted to the case where R_1 may not always be 1. If mortal-cohort NFD holds, the selection bias function and the sensitivity parameter γ are correctly chosen, and if either the dropout models are correctly specified at all time points or the outcome regression models are correctly specified at all time points, then the estimator $\hat{\beta}$ that solves

$$\Psi\left(\hat{\alpha}, \hat{\theta}, \gamma, \beta\right) = \sum_{i=1}^{n}\sum_{t=1}^{J} A_{it}\left(\frac{\partial \mu_{it}}{\partial \beta}\right) \times \left[\frac{R_{it}(Y_{it} - \mu_{it})}{\lambda_{it}(\bar{O}_{i,t-1}, Y_{it}; \hat{\alpha}_t, \gamma)} + \sum_{l=0}^{t-1} \frac{R_{il}}{\lambda_{il}(\bar{O}_{i,l-1}, Y_{il}; \hat{\alpha}_l, \gamma)}\left\{1 - \frac{R_{i,l+1}}{\pi_{i,l+1}(\bar{O}_{il}, Y_{i,l+1}; \hat{\alpha}_{l+1}, \gamma)}\right\}\left\{m_t(\bar{O}_{il}; \hat{\theta}_{t,l}) - \mu_{it}\right\}\right] = 0 \quad \text{(E.1)}$$

is consistent.

We must first prove the following Lemma 4. For the proof, we suppress the subscript i, let $m_t(\bar{O}_l) = m_t(\bar{O}_l; \theta_{t,l})$, let $\pi_t = \pi_t(\bar{O}_{t-1}, Y_t; \alpha_t, \gamma)$ and let $\lambda_t = \lambda_t(\bar{O}_{t-1}, Y_t; \alpha, \gamma)$.

Lemma 4: *The expression in the large parenthesis $[\cdot]$ in equation (E.1) can be written as the following:*

$$R_t(Y_t - \mu_t) + \sum_{l=1}^{t-1} R_l(1 - R_{l+1})\{m_t(\bar{O}_l) - \mu_t\} + \frac{R_t}{\lambda_{t-1}}\left(\frac{1-\pi_t}{\pi_t}\right)\left\{Y_t - m_t\left(\bar{O}_{t-1}\right)\right\} + \sum_{l=1}^{t-2}\frac{R_{l+1}}{\lambda_l}\left(\frac{1-\pi_{l+1}}{\pi_{l+1}}\right)\left\{R_tY_t + \sum_{k=l+1}^{t-1} R_k(1 - R_{k+1})m_t(\bar{O}_k) - m_t(\bar{O}_l)\right\} \quad \text{(E.2)}$$

Proof. (Lemma 4) We prove this Lemma by showing that (E.2) can be rewritten as (E.1). Combining like terms, (E.2) can be re-written as the sum of:

(1) $\left\{R_t + \sum_{l=1}^{t-1}\frac{R_t}{\lambda_l}\left(\frac{1-\pi_{l+1}}{\pi_{l+1}}\right)\right\} Y_t$

(2) $\displaystyle\sum_{s=1}^{t-1}\left\{R_s(1 - R_{s+1}) + \sum_{l=1}^{s-1}\frac{R_s(1-R_{s+1})}{\lambda_l}\left(\frac{1-\pi_{l+1}}{\pi_{l+1}}\right) - \frac{R_{s+1}}{\lambda_s}\left(\frac{1-\pi_{s+1}}{\pi_{s+1}}\right)\right\} m_t\left(\bar{O}_s\right)$

(3) $\left\{-R_t - \sum_{l=1}^{t-1} R_l(1 - R_{l+1})\right\}\mu_t$

for each t. Now we can show that each item can be re-written and combined to give the expression in $[\cdot]$ of equation (E.1).

For term (1):

$$\begin{aligned}\left\{R_t + \sum_{l=1}^{t-1} \frac{R_t}{\lambda_l}\left(\frac{1-\pi_{l+1}}{\pi_{l+1}}\right)\right\} Y_t &= \left\{R_t + R_t \sum_{l=1}^{t-1} \frac{1}{\lambda_l}\left(\frac{1-\pi_{l+1}}{\pi_{l+1}}\right)\right\} Y_t \\ &= \left\{R_t + R_t\left[\left(\frac{1-\pi_2}{\pi_2}\right) + \frac{1}{\lambda_2}\left(\frac{1-\pi_3}{\pi_3}\right) + \sum_{l=3}^{t-1}\frac{1}{\lambda_l}\left(\frac{1-\pi_{l+1}}{\pi_{l+1}}\right)\right]\right\} Y_t \\ &= \left\{R_t + R_t\left[\left(\frac{1-\lambda_3}{\lambda_3}\right) + \sum_{l=3}^{t-1}\frac{1}{\lambda_l}\left(\frac{1-\pi_{l+1}}{\pi_{l+1}}\right)\right]\right\} Y_t \qquad \text{(E.3)} \\ &= \left\{R_t + R_t\left(\frac{1-\lambda_t}{\lambda_t}\right)\right\} Y_t \qquad \text{(E.4)} \\ &= \left(\frac{R_t}{\lambda_t}\right) Y_t\end{aligned}$$

where equation (E.4) can be derived from equation (E.3) using induction.

For term (2):

$$\begin{aligned}&\left\{R_s(1-R_{s+1}) + \sum_{l=1}^{s-1}\frac{R_s(1-R_{s+1})}{\lambda_l}\left(\frac{1-\pi_{l+1}}{\pi_{l+1}}\right) - \frac{R_{s+1}}{\lambda_s}\left(\frac{1-\pi_{s+1}}{\pi_{s+1}}\right)\right\} m_t\left(\bar{O}_s\right),\ \forall s \leqslant t-1 \\ &= \left\{\frac{R_s(1-R_{s+1})\lambda_{s+1} - R_{s+1}(1-\pi_{s+1})}{\lambda_{s+1}} + R_s(1-R_{s+1})\sum_{l=1}^{s-1}\frac{1}{\lambda_l}\left(\frac{1-\pi_{l+1}}{\pi_{l+1}}\right)\right\} m_t\left(\bar{O}_s\right) \\ &= \left\{\frac{R_s(1-R_{s+1})\lambda_{s+1} - R_{s+1}(1-\pi_{s+1})}{\lambda_{s+1}} + R_s(1-R_{s+1})\left[\frac{1-\pi_2}{\pi_2} + \frac{1}{\lambda_2}\left(\frac{1-\pi_3}{\pi_3}\right) + \sum_{l=3}^{s-1}\frac{1}{\lambda_l}\left(\frac{1-\pi_{l+1}}{\pi_{l+1}}\right)\right]\right\} m_t\left(\bar{O}_s\right) \\ &= \left\{\frac{R_s(1-R_{s+1})\lambda_{s+1} - R_{s+1}(1-\pi_{s+1})}{\lambda_{s+1}} + R_s(1-R_{s+1})\left[\frac{1-\lambda_3}{\lambda_3} + \sum_{l=3}^{s-1}\frac{1}{\lambda_l}\left(\frac{1-\pi_{l+1}}{\pi_{l+1}}\right)\right]\right\} m_t\left(\bar{O}_s\right) \qquad \text{(E.5)} \\ &= \left\{\frac{R_s(1-R_{s+1})\lambda_{s+1} - R_{s+1}(1-\pi_{s+1})}{\lambda_{s+1}} + R_s(1-R_{s+1})\left(\frac{1-\lambda_s}{\lambda_s}\right)\right\} m_t\left(\bar{O}_s\right) \qquad \text{(E.6)} \\ &= \left\{\frac{R_s(1-R_{s+1})\lambda_{s+1} + R_s(1-R_{s+1})(1-\lambda_s)\pi_{s+1}}{\lambda_{s+1}} - \frac{R_{s+1}(1-\pi_{s+1})}{\lambda_{s+1}}\right\} m_t\left(\bar{O}_s\right) \\ &= \left\{\frac{R_s\pi_{s+1} - R_{s+1}}{\lambda_{s+1}}\right\} m_t\left(\bar{O}_s\right) \\ &= \frac{R_s}{\lambda_s}\left(1 - \frac{R_{s+1}}{\pi_{s+1}}\right) m_t\left(\bar{O}_s\right)\end{aligned}$$

where equation (E.6) can be derived from equation (E.5) using induction.

Finally, for term (3):

$$\begin{aligned}\left\{-R_t - \sum_{l=1}^{t-1} R_l(1-R_{l+1})\right\}\mu_t &= -\left\{R_t + \sum_{l=1}^{t-1}(R_l - R_{l+1})\right\}\mu_t \\ &= -\left\{R_t + R_{t-1} - R_t + R_{t-2} - R_{t-1} + \ldots + R_1 - R_2\right\}\mu_t \qquad \text{(E.7)} \\ &= -\mu_t \\ &= -\left\{\frac{R_t}{\lambda_t} + \frac{R_{t-1}}{\lambda_{t-1}} - \frac{R_t}{\lambda_t} + \frac{R_{t-2}}{\lambda_{t-2}} - \frac{R_{t-1}}{\lambda_{t-1}} + \ldots + \frac{R_1}{\lambda_1} - \frac{R_2}{\lambda_2}\right\}\mu_t \qquad \text{(E.8)}\end{aligned}$$

$$= -\left\{\frac{R_t}{\lambda_t} + \sum_{l=1}^{t-1}\left(\frac{R_l}{\lambda_l} - \frac{R_{l+1}}{\lambda_{l+1}}\right)\right\}\mu_t$$
$$= -\left\{\frac{R_t}{\lambda_t} + \sum_{l=1}^{t-1}\frac{R_l}{\lambda_l}\left(1 - \frac{R_{l+1}}{\pi_{l+1}}\right)\right\}\mu_t$$

Taking the sums of the expressions (1), (2) and (3) would result in the expression in the large parenthesis $[\cdot]$ in equation (E.1) as required.

Proof. (Unbiasedness of AIPW estimating equations)

For simplicity, we assume that X is a vector of time-independent auxiliary variables that include Z. Let the true values of α and θ be represented by α_0 and θ_0. Let $\hat{\alpha}$ and $\hat{\theta}$ be estimators of α and θ, and let α^* and θ^* be the probability limits of $\hat{\alpha}$ and $\hat{\theta}$. In addition, let $\pi_t^* = \pi_t^*(\bar{O}_{j-1}, Y_j) = \pi(\bar{O}_{j-1}, Y_j; \alpha_t^*, \gamma)$, let $\lambda_t^* = \lambda_t^*(\bar{O}_{t-1}, Y_t) = \lambda(\bar{O}_{t-1}, Y_t; \alpha^*, \gamma)$ and let $m_t^*(\bar{O}_l) = m_t\left(\bar{O}_l; \theta_{t,l}^*\right)$.

Let the true value of β be β_0. To show double robustness, we show that when $\alpha^* = \alpha_0$ or when $\theta^* = \theta_0$, then $E\{\Psi(\alpha^*, \theta^*, \gamma, \beta_0)\} = 0$. First we assume that the dropout models are correctly specified at all visits t and that $\alpha^* = \alpha_0$. We show that the estimating equations in equation (E.1) have expectation zero.

Take expectation with respect to the first term of equation (E.1):

$$E_{Y_t|\bar{Y}_{t-1},X,A_t=1} E_{\bar{R}_t|\bar{Y}_t,X A_t=1}\left\{\frac{R_t(Y_t - \mu_t)}{\lambda_t}\right\} = 0$$

Now take expectation with respect to the second term of equation (E.1):

$$E_{\bar{R}_l,\bar{Y}_{l+1}|X,A_t=1} E_{R_{l+1}|\bar{R}_l,\bar{Y}_{l+1},X,A_t=1}\left[\frac{R_l}{\lambda_l^*}\left(1 - \frac{R_{l+1}}{\pi_{l+1}^*}\right)\left\{m_t^*\left(\bar{O}_l\right) - \mu_t\right\}\right]$$
$$= E_{\bar{R}_l,\bar{Y}_{l+1}|X,A_t=1}\left[\left\{\frac{R_l}{\lambda_l^*}\left(1 - \frac{1}{\pi_{l+1}^*}\right)\pi_{l+1}^* + \frac{R_l}{\lambda_l^*}\left(1 - \pi_{l+1}^*\right)\right\}\left\{m_t^*\left(\bar{O}_l\right) - \mu_t\right\}\right]$$
$$= E_{\bar{R}_l,\bar{Y}_{l+1}|X,A_t=1}\left[\frac{R_l}{\lambda_l^*}\left[\left(\pi_{l+1}^* - 1\right) + \left(1 - \pi_{l+1}^*\right)\right]\left\{m_t^*\left(\bar{O}_l\right) - \mu_t\right\}\right]$$
$$= 0$$

Next we assume that the outcome regression models are correctly specified at all visits t and that $\theta_0 = \theta^*$. We show that the estimating equations in equation (E.1) with the content in the

large parenthesis [·] replaced by equation (E.2) have expectation zero. For the proof, we break the items in equation (E.2) into sections, and let $h_{0t} = P(R_t = 1 \mid R_{t-1} = 1, \bar{Y}_{t-1}, X, A_t = 1)$.

First two terms of equation (E.2): $R_t(Y_t - \mu_t) + \sum_{l=1}^{t-1} R_l(1 - R_{l+1})[m_t(\bar{O}_l) - \mu_t\}$

$$
\begin{aligned}
& E_{\bar{Y}_{t-1},\bar{R}_t|X,A_t=1} E_{Y_t|\bar{Y}_{t-1},\bar{R}_t,X,A_t=1}\left[R_t(Y_t - \mu_t) + \sum_{l=1}^{t-1} R_l(1 - R_{l+1})\{m_t^*(\bar{O}_l) - \mu_t\}\right] \\
&= E_{\bar{Y}_{t-1},\bar{R}_{t-1}|X,A_t=1} E_{R_t|\bar{Y}_{t-1},\bar{R}_{t-1},X,A_t=1}\left[R_t\left\{E(Y_t \mid \bar{Y}_{t-1}, \bar{R}_t, X, A_t = 1) - \mu_t\right\} + \sum_{l=1}^{t-1} R_l(1 - R_{l+1})\left\{m_t^*(\bar{O}_l) - \mu_t\right\}\right] \\
&= E_{\bar{Y}_{t-1},\bar{R}_{t-1}|X,A_t=1}\Bigg[h_{0t}R_{t-1}\left\{E_{Y_t}(Y_t \mid \bar{Y}_{t-1}, R_t = 1, X, A_t = 1) - \mu_t\right\} + (1 - h_{0t})R_{t-1}\left\{m_t^*(\bar{O}_{t-1}) - \mu_t\right\} + \\
&\qquad \sum_{l=1}^{t-2} R_l(1 - R_{l+1})\left\{m_t^*(\bar{O}_l) - \mu_t\right\}\Bigg] \\
&= E_{\bar{Y}_{t-1},\bar{R}_{t-1}|X,A_t=1}\left[R_{t-1}\left\{E_{Y_t}(Y_t \mid \bar{Y}_{t-1}, R_{t-1} = 1, X, A_t = 1) - \mu_t\right\} + \sum_{l=1}^{t-2} R_l(1 - R_{l+1})\left\{m_t^*(\bar{O}_l) - \mu_t\right\}\right] \\
&= E_{\bar{R}_{t-1},\bar{Y}_{t-2}|X,A_t=1} E_{Y_{t-1}|\bar{Y}_{t-2},\bar{R}_{t-1},X,A_t=1}\Bigg[R_{t-1}\left\{E_{Y_t}(Y_t \mid \bar{Y}_{t-1}, R_{t-1} = 1, X, A_t = 1) - \mu_t\right\} + \\
&\qquad \sum_{l=1}^{t-2} R_l(1 - R_{l+1})\left\{m_t^*(\bar{O}_l) - \mu_t\right\}\Bigg] \\
&= E_{\bar{R}_{t-1},\bar{Y}_{t-2}|X,A_t=1}\Bigg(R_{t-1}\Big[E_{Y_{t-1}}\Big\{E_{Y_t}(Y_t \mid \bar{Y}_{t-1}, R_{t-1} = 1, X, A_t = 1)\Big|\bar{Y}_{t-2}, \bar{R}_{t-1}, X, A_t = 1\Big\} - \mu_t\Big] + \\
&\qquad \sum_{l=1}^{t-2} R_l(1 - R_{l+1})\left\{m_t^*(\bar{O}_l) - \mu_t\right\}\Bigg) \\
&= E_{\bar{R}_{t-2},\bar{Y}_{t-2}|X,A_t=1} E_{R_{t-1}|\bar{Y}_{t-2},\bar{R}_{t-2},X,A_t=1}\Bigg(R_{t-1}\Big[E_{Y_{t-1}}\Big\{E_{Y_t}(Y_t \mid \bar{Y}_{t-1}, R_{t-1} = 1, X, A_t = 1)\Big|\bar{Y}_{t-2}, \bar{R}_{t-1}, X, A_t = 1\Big\} - \\
&\qquad \mu_t\Big] + \sum_{l=1}^{t-2} R_l(1 - R_{l+1})\left\{m_t^*(\bar{O}_l) - \mu_t\right\}\Bigg) \\
&= E_{\bar{R}_{t-2},\bar{Y}_{t-2}|X,A_t=1}\Bigg(h_{0,t-1}R_{t-2}\Big[E_{Y_{t-1}}\Big\{E_{Y_t}(Y_t \mid \bar{Y}_{t-1}, R_{t-1} = 1, X, A_t = 1)\Big|\bar{Y}_{t-2}, R_{t-1} = 1, X, A_t = 1\Big\} - \mu_t\Big] + \\
&\qquad (1 - h_{0,t-1})R_{t-2}\left\{m_t^*(\bar{O}_{t-2}) - \mu_t\right\} + \sum_{l=1}^{t-3} R_l(1 - R_{l+1})\left\{m_t^*(\bar{O}_l) - \mu_t\right\}\Bigg) \\
&= E_{\bar{Y}_{t-2},\bar{R}_{t-2}|X,A_t=1}\left[R_{t-2}\left\{E_{Y_t}(Y_t \mid \bar{Y}_{t-2}, R_{t-2} = 1, X, A_t = 1) - \mu_t\right\} + \sum_{l=1}^{t-3} R_l(1 - R_{l+1})\left\{m_t^*(\bar{O}_l) - \mu_t\right\}\right] \qquad \text{(E.9)}
\end{aligned}
$$

Using induction by taking iterative expectations

$$
E_{R_2|\bar{Y}_1,\bar{R}_1,X,A_t=1} E_{Y_2|\bar{Y}_1,\bar{R}_2,X,A_t=1} \ldots E_{R_{t-2}|\bar{Y}_{t-3},\bar{R}_{t-3},X,A_t=1} E_{Y_{t-2}|\bar{Y}_{t-3},\bar{R}_{t-2},X,A_t=1}(\,\cdot\,)
$$

on equation (E.9), we obtain:

$$
\begin{aligned}
& E_{Y_1|A_t=1,X}\Big(h_{02}\left[E_{Y_2}\left\{E_{Y_t}(Y_t|\bar{Y}_2, R_2 = 1, A_t = 1, X) \mid Y_1, R_2 = 1, A_t = 1, X\right\} - \mu_t\right] + \left\{m_t^*(\bar{O}_1) - \mu_t\right\}(1 - h_{02})\Big) \\
&= E_{Y_1|A_t=1,X}\left\{E_{Y_t}\left(Y_t|Y_1, A_t = 1, X\right) - \mu_t)\right\} = 0
\end{aligned}
$$

Third term of equation (E.2): $\frac{R_t}{\lambda_{t-1}}\left(\frac{1-\pi_t}{\pi_t}\right)\left\{Y_t - m_t\left(\bar{O}_{t-1}\right)\right\}$

$$
\begin{aligned}
& E_{\bar{Y}_t,\bar{R}_t|A_t=1,X}\left[\frac{R_t}{\lambda_{t-1}}\left(\frac{1-\pi_t}{\pi_t}\right)\left\{Y_t - m_t^*\left(\bar{O}_{t-1}\right)\right\}\right] \\
&= E_{\bar{R}_t,\bar{Y}_{t-1}|A_t=1,X}E_{Y_t|\bar{Y}_{t-1},\bar{R}_t,A_t=1,X}\left[\frac{R_t}{\lambda_{t-1}}\left(\frac{1-\pi_t}{\pi_t}\right)\left\{Y_t - m_t^*\left(\bar{O}_{t-1}\right)\right\}\right] \\
&= E_{\bar{Y}_{t-1}|A_t=1,X}E_{\bar{R}_t|\bar{Y}_{t-1},A_t=1,X}E_{Y_t|\bar{Y}_{t-1},\bar{R}_t,A_t=1,X}\left[\frac{R_t}{\lambda_{t-1}}g^*\left(\bar{O}_{t-1}\right)e^{q_t(\bar{O}_{t-1},Y_t)}\left\{Y_t - m_t^*\left(\bar{O}_{t-1}\right)\right\}\right] \\
&= E_{\bar{Y}_{t-1}|A_t=1,X}\left[\frac{1}{\lambda_{t-1}}g^*\left(\bar{O}_{t-1}\right)\left\{E\left(Y_t e^{q_t(\bar{O}_{t-1},Y_t)}|\bar{Y}_{t-1},R_t=1,A_t=1,X\right) - \right.\right. \\
&\qquad \left.\left. E\left(e^{q_t(\bar{O}_{t-1},Y_t)}|\bar{Y}_{t-1},R_t=1,A_t=1,X\right)m_t^*\left(\bar{O}_{t-1}\right)\right\}P(R_t=1|\bar{Y}_{t-1},A_t=1,X)\right] \\
&= 0 \text{ (By Lemma 1)}
\end{aligned}
$$

Here $g^*\left(\bar{O}_{t-1}\right)$ is defined by the postulated dropout model at visit t.

Fourth term of equation (E.2): $\sum_{l=1}^{t-2}\frac{R_{l+1}}{\lambda_l}\left(\frac{1-\pi_{l+1}}{\pi_{l+1}}\right)\left\{R_tY_t + \sum_{k=l+1}^{t-1}R_k(1-R_{k+1})m_t(\bar{O}_k) - m_t(\bar{O}_l)\right\}$

The two lines of the proof is similar to the proof for the first two terms of equation (E.2). It can be shown that

$$
\begin{aligned}
& E_{\bar{R}_{l+1},\bar{Y}_{l+1}|A_t=1,X}E_{R_{l+2},\ldots,R_t,Y_{l+2},Y_t|\bar{R}_{l+1},\bar{Y}_{l+1},A_t=1,X}\left[\sum_{l=1}^{t-2}\frac{R_{l+1}}{\lambda_l}\left(\frac{1-\pi_{l+1}}{\pi_{l+1}}\right)\left\{R_tY_t + \right.\right. \\
&\qquad \left.\left.\sum_{k=l+1}^{t-1}R_k(1-R_{k+1})m_t^*(\bar{O}_k) - m_t^*(\bar{O}_l)\right\}\right] \\
&= E_{\bar{R}_{l+1},\bar{Y}_{l+1}|A_t=1,X}\left[\sum_{l=1}^{t-2}\frac{R_{l+1}}{\lambda_l}\left(\frac{1-\pi_{l+1}}{\pi_{l+1}}\right)\left\{E_{Y_t}(Y_t|\bar{Y}_{l+1},R_{l+1}=1,A_t=1,X) - m_t^*(\bar{O}_l)\right\}\right] \\
&= E_{\bar{R}_{l+1},\bar{Y}_{l+1}|A_t=1,X}\left[\sum_{l=1}^{t-2}\frac{R_{l+1}}{\lambda_l}g^*(\bar{O}_l)e^{q_{l+1}(\bar{O}_l,Y_{l+1})}\left\{E_{Y_t}(Y_t|\bar{Y}_{l+1},R_{l+1}=1,A_t=1,X) - m_t^*(\bar{O}_l)\right\}\right] \\
&= E_{\bar{Y}_l|A_t=1,X}E_{R_{l+1}|\bar{Y}_l,A_t=1,X}E_{Y_{l+1}|\bar{R}_{l+1},\bar{Y}_l,A_t=1,X}\left[\sum_{l=1}^{t-2}\frac{R_{l+1}}{\lambda_l}g^*(\bar{O}_l)e^{q_{l+1}(\bar{O}_l,Y_{l+1})}\times\right. \\
&\qquad \left.\left\{E_{Y_t}(Y_t|\bar{Y}_{l+1},R_{l+1}=1,A_t=1,X) - m_t^*(\bar{O}_l)\right\}\right] \\
&= E_{\bar{Y}_l|A_t=1,X}\left[\sum_{l=1}^{t-2}\frac{1}{\lambda_l}g^*(\bar{O}_l)\left\{E_{Y_{l+1}}\left(e^{q_{l+1}(\bar{O}_l,Y_{l+1})}E_{Y_t}(Y_t|\bar{Y}_{l+1},R_{l+1}=1,A_t=1,X)\Big|R_{l+1}=1,\bar{Y}_l,A_t=1,X\right) - \right.\right. \\
&\qquad \left.\left. E_{Y_{l+1}}\left(e^{q_{l+1}(\bar{O}_l,Y_{l+1})}|R_{l+1}=1,\bar{Y}_l,A_t=1,X\right)m_t^*\left(\bar{O}_l\right)\right\}P(R_{l+1}=1|\bar{Y}_{l-1},A_t=1,X)\right] \\
&= 0 \text{ (By Lemma 2)}
\end{aligned}
$$

Appendix F

Proof of equivalence

We prove that when $q_t(\bar{O}_{t-1}, Y_t) = 0$ and D is included in both of the dropout and outcome regression models, the AIPW estimator in Wen *and others* (2017) (Theorem 2) is the same as the one in the current paper for monotone missing data. Like Wen *and others* (2017), we suppose that $R_1 = 1$. When $q_t(\bar{O}_{t-1}, Y_t) = 0$, then for all $s < t \leqslant D$,

$$E(Y_t \mid \bar{O}_s, R_s = 1, R_{s+1} = 0, D) = E(Y_t \mid \bar{O}_s, Y_s, R_s = 1, D)$$

Let $U(\beta) = U(\bar{Y}_D, D, Z; \beta) = \sum_{t=1}^{D} Z_t(Y_t - \mu_t)$ and suppose that $\dfrac{\partial \mu_t}{\partial \beta} = Z_t$. Let $H_l(\bar{O}_l, D; \beta, \gamma)$ be a model for for $E\left\{U(\beta) \mid \bar{O}_l, D, R_l = 1\right\}$ with parameters β and γ, and let $\lambda_l(\bar{O}_{l-1}, D; \alpha)$ be a model for $P(R_l = 1|\bar{O}_{l-1}, D)$ with parameters α. In addition, let $\pi_l(\bar{O}_{l-1}, D; \alpha_l)$ be a model for $P(R_l = 1|R_{l-1} = 1, \bar{O}_{l-1}, D)$. We suppress the parameters for the proof.

A subject's contribution to the AIPW estimator in Wen *and others* (2017) is given by

$$\frac{R_D}{\lambda_D(\bar{O}_{D-1}, D)} U(\beta) + \sum_{l=1}^{D-1} \left\{ \frac{R_l}{\lambda_l(\bar{O}_{l-1}, D)} - \frac{R_{l+1}}{\lambda_{l+1}(\bar{O}_l, D)} \right\} H_l(\bar{O}_l, D) \tag{F.1}$$

When $q_t(\bar{O}_{t-1}, Y_t) = 0$, we let $s_t(\bar{O}_s, D; \gamma_{t,l})$ be a model for $E(Y_t \mid \bar{O}_s, Y_s, R_s = 1, D)$ with parameters γ. A subject's contribution (suppressing parameter) to the AIPW estimator in the current paper is given by

$$\sum_{t=1}^{J} A_t Z_t \left[\frac{R_t(Y_t - \mu_t)}{\lambda_t(\bar{O}_{t-1}, D)} + \sum_{l=1}^{t-1} \frac{R_l}{\lambda_l(\bar{O}_{l-1}, D)} \left\{ 1 - \frac{R_{l+1}}{\pi_{l+1}(\bar{O}_l, D)} \right\} \left\{ s_t(\bar{O}_l, D) - \mu_t \right\} \right] \tag{F.2}$$

When $D = 1$, equivalence is not difficult to prove. For any $D > 1$ (i.e. $D = 1, 2, \ldots, J$), the estimating equations given in (F.2) is then

$$\sum_{t=1}^{D} Z_t \frac{R_t(Y_t - \mu_t)}{\lambda_t(\bar{O}_{t-1}, D)} + \sum_{t=1}^{D} Z_t \left[\sum_{l=1}^{t-1} \frac{R_l}{\lambda_l(\bar{O}_{l-1}, D)} \left\{ 1 - \frac{R_{l+1}}{\pi_{l+1}(\bar{O}_l, D)} \right\} \left\{ s_t(\bar{O}_l, D) - \mu_t \right\} \right] \tag{F.3}$$

given that $\sum_{t=1}^{0}\{\cdot\} = 1$.

The estimating equations given in (F.1) is then

$$\frac{R_D}{\lambda_D(\bar{O}_{D-1}, D)} \sum_{t=1}^{D} Z_t(Y_t - \mu_t) + \sum_{l=1}^{D-1} \left\{ \frac{R_l}{\lambda_l(\bar{O}_{l-1}, D)} - \frac{R_{l+1}}{\lambda_{l+1}(\bar{O}_l, D)} \right\} H_l(\bar{O}_l, D)$$

which can be written as

$$
\begin{aligned}
& Z_D \frac{R_D(Y_D-\mu_D)}{\lambda_D(\bar{O}_{D-1},D)} + \left\{\frac{R_{D-1}}{\lambda_{D-1}(\bar{O}_{D-2},D)} - \frac{R_D}{\lambda_D(\bar{O}_{D-1},D)}\right\} E\left\{\sum_{t=1}^{D} Z_t(Y_t-\mu_t) \mid \bar{O}_{D-1}, R_{D-1}=1, D\right\} + \\
& \quad \frac{R_D}{\lambda_D(\bar{O}_{D-1},D)} \sum_{t=1}^{D-1} Z_t(Y_t-\mu_t) + \sum_{l=1}^{D-2}\left\{\frac{R_l}{\lambda_l(\bar{O}_{l-1},D)} - \frac{R_{l+1}}{\lambda_{l+1}(\bar{O}_l,D)}\right\} E\left\{\sum_{t=1}^{D} Z_t(Y_t-\mu_t) \mid \bar{O}_l, R_l=1, D\right\} \\
& = Z_D \frac{R_D(Y_D-\mu_D)}{\lambda_D(\bar{O}_{D-1},D)} + Z_D\left\{\frac{R_{D-1}}{\lambda_{D-1}(\bar{O}_{D-2},D)} - \frac{R_D}{\lambda_D(\bar{O}_{D-1},D)}\right\}\{s_D(\bar{O}_{D-1},D)-\mu_D\} + \\
& \quad \left\{\frac{R_{D-1}}{\lambda_{D-1}(\bar{O}_{D-2},D)} - \frac{R_D}{\lambda_D(\bar{O}_{D-1},D)}\right\} \sum_{t=1}^{D-1} Z_t(Y_t-\mu_t) + \frac{R_D}{\lambda_D(\bar{O}_{D-1},D)} \sum_{t=1}^{D-1} Z_t(Y_t-\mu_t) + \\
& \quad \sum_{l=1}^{D-2}\left\{\frac{R_l}{\lambda_l(\bar{O}_{l-1},D)} - \frac{R_{l+1}}{\lambda_{l+1}(\bar{O}_l,D)}\right\} E\left\{\sum_{t=1}^{D} Z_t(Y_t-\mu_t) \mid \bar{O}_l, R_l=1, D\right\} \\
& = Z_D \frac{R_D(Y_D-\mu_D)}{\lambda_D(\bar{O}_{D-1},D)} + Z_D\left\{\frac{R_{D-1}}{\lambda_{D-1}(\bar{O}_{D-2},D)} - \frac{R_D}{\lambda_D(\bar{O}_{D-1},D)}\right\}\{s_D(\bar{O}_{D-1},D)-\mu_D\} + \\
& \quad \frac{R_{D-1}}{\lambda_{D-1}(\bar{O}_{D-2},D)} \sum_{t=1}^{D-1} Z_t(Y_t-\mu_t) + \sum_{l=1}^{D-2}\left\{\frac{R_l}{\lambda_l(\bar{O}_{l-1},D)} - \frac{R_{l+1}}{\lambda_{l+1}(\bar{O}_l,D)}\right\} E\left\{\sum_{t=1}^{D} Z_t(Y_t-\mu_t) \mid \bar{O}_l, R_l=1, D\right\} \\
& = Z_D \frac{R_D(Y_D-\mu_D)}{\lambda_D(\bar{O}_{D-1},D)} + Z_D\left\{\frac{R_{D-1}}{\lambda_{D-1}(\bar{O}_{D-2},D)} - \frac{R_D}{\lambda_D(\bar{O}_{D-1},D)}\right\}\{s_D(\bar{O}_{D-1},D)-\mu_D\} + \\
& \quad \frac{R_{D-1}}{\lambda_{D-1}(\bar{O}_{D-2},D)} \sum_{t=1}^{D-1} Z_t(Y_t-\mu_t) + \left\{\frac{R_{D-2}}{\lambda_{D-2}(\bar{O}_{D-3},D)} - \frac{R_{D-1}}{\lambda_{D-1}(\bar{O}_{D-2},D)}\right\} E\left\{\sum_{t=1}^{D} Z_t(Y_t-\mu_t) \mid \bar{O}_{D-2}, R_{D-2}=1, D\right\} + \\
& \quad \sum_{l=1}^{D-3}\left\{\frac{R_l}{\lambda_l(\bar{O}_{l-1},D)} - \frac{R_{l+1}}{\lambda_{l+1}(\bar{O}_l,D)}\right\} E\left\{\sum_{t=1}^{D} Z_t(Y_t-\mu_t) \mid \bar{O}_l, R_l=1, D\right\} \\
& = Z_D \frac{R_D(Y_D-\mu_D)}{\lambda_D(\bar{O}_{D-1},D)} + Z_D\left\{\frac{R_{D-1}}{\lambda_{D-1}(\bar{O}_{D-2},D)} - \frac{R_D}{\lambda_D(\bar{O}_{D-1},D)}\right\}\{s_D(\bar{O}_{D-1},D)-\mu_D\} + Z_{D-1}\frac{R_{D-1}(Y_{D-1}-\mu_{D-1})}{\lambda_{D-1}(\bar{O}_{D-2},D)} + \\
& \quad \frac{R_{D-1}}{\lambda_{D-1}(\bar{O}_{D-2},D)} \sum_{t=1}^{D-2} Z_t(Y_t-\mu_t) + \left\{\frac{R_{D-2}}{\lambda_{D-2}(\bar{O}_{D-3},D)} - \frac{R_{D-1}}{\lambda_{D-1}(\bar{O}_{D-2},D)}\right\} E\left\{\sum_{t=D-1}^{D} Z_t(Y_t-\mu_t) \mid \bar{O}_{D-2}, R_{D-2}=1, D\right\} + \\
& \quad \left\{\frac{R_{D-2}}{\lambda_{D-2}(\bar{O}_{D-3},D)} - \frac{R_{D-1}}{\lambda_{D-1}(\bar{O}_{D-2},D)}\right\} \sum_{t=1}^{D-2} Z_t(Y_t-\mu_t) + \\
& \quad \sum_{l=1}^{D-3}\left\{\frac{R_l}{\lambda_l(\bar{O}_{l-1},D)} - \frac{R_{l+1}}{\lambda_{l+1}(\bar{O}_l,D)}\right\} E\left\{\sum_{t=1}^{D} Z_t(Y_t-\mu_t) \mid \bar{O}_l, R_l=1, D\right\} \\
& = \sum_{t=D-2}^{D} Z_t \frac{R_t(Y_t-\mu_t)}{\lambda_t(\bar{O}_{t-1},D)} + \sum_{t=D-1}^{D} Z_t \left[\sum_{l=D-2}^{t-1} \frac{R_l}{\lambda_l(\bar{O}_{l-1},D)}\left\{1-\frac{R_{l+1}}{\pi_{l+1}(\bar{O}_l,D)}\right\}\{s_t(\bar{O}_l,D)-\mu_t\}\right] + \\
& \quad \frac{R_{D-2}}{\lambda_{D-2}(\bar{O}_{D-3},D)} \sum_{t=1}^{D-3} Z_t(Y_t-\mu_t) + \sum_{l=1}^{D-3}\left\{\frac{R_l}{\lambda_l(\bar{O}_{l-1},D)} - \frac{R_{l+1}}{\lambda_{l+1}(\bar{O}_l,D)}\right\} E\left\{\sum_{t=1}^{D} Z_t(Y_t-\mu_t) \mid \bar{O}_l, R_l=1, D\right\} \\
& = \sum_{t=2}^{D} Z_t \frac{R_t(Y_t-\mu_t)}{\lambda_t(\bar{O}_{t-1},D)} + \sum_{t=3}^{D} Z_t \left[\sum_{l=2}^{t-1} \frac{R_l}{\lambda_l(\bar{O}_{l-1},D)}\left\{1-\frac{R_{l+1}}{\pi_{l+1}(\bar{O}_l,D)}\right\}\{s_t(\bar{O}_l,D)-\mu_t\}\right] + \qquad \text{(F.4)} \\
& \quad \frac{R_2}{\lambda_2(\bar{O}_1,D)} Z_1(Y_1-\mu_1) + \left\{1-\frac{R_2}{\lambda_2}\right\} E\left\{\sum_{t=1}^{D} Z_t(Y_t-\mu_t) \mid \bar{O}_1, R_1=1, D\right\} \\
& = \sum_{t=1}^{D} Z_t \frac{R_t(Y_t-\mu_t)}{\lambda_t(\bar{O}_{t-1},D)} + \sum_{t=2}^{D} Z_t \left[\sum_{l=1}^{t-1} \frac{R_l}{\lambda_l(\bar{O}_{l-1},D)}\left\{1-\frac{R_{l+1}}{\pi_{l+1}(\bar{O}_l,D)}\right\}\{s_t(\bar{O}_l,D)-\mu_t\}\right]
\end{aligned}
$$

where equation (F.4) is derived from induction. The last line is equivalent to equation (F.3) by the definition that $\sum_{t=1}^{0}\{\cdot\}=1$ (as given in Appendix E).

Appendix G

Asymptotic distribution of the AIPW estimator in monotone missing data

Let α^* and θ^* be the probability limits of $\hat{\alpha}$ and $\hat{\theta}$, where α^* and θ^* are not necessarily equal to α_0 and θ_0. In general, α^* or θ^* only equals α_0 or θ_0 when the model for $\pi_t(\bar{O}_{t-1}, Y_t)$ or $m_t(\bar{O}_l)$, respectively, is correctly specified. Let $Q(\alpha) = \left(Q_1(\alpha_1)', \ldots, Q_J(\alpha_J)'\right)'$ and $M(\theta) = \left(M_1(\theta_{1,0})', M_2(\theta_{2,1})', \ldots, M_J(\theta_{J,J-1})'\right)'$.

THEOREM 2: *Suppose the regularity conditions given below hold. Then*

$$\sqrt{n}(\hat{\beta} - \beta_0) \longrightarrow MVN\left\{0, \Sigma_{\Psi_\beta}^{-1}\Omega_\Psi(\Sigma_{\Psi_\beta}^{-1})^T\right\} \tag{G.1}$$

where

$$\Omega_\Psi = E\left[\left\{\Psi(\alpha^*, \theta^*, \gamma, \beta_0) - A_{\Psi_\alpha}B_Q^{-1}Q(\alpha^*) - A_{\Psi_\theta}B_M^{-1}M(\theta^*)\right\}^{\otimes 2}\right]$$

$$\Sigma_{\Psi_\beta} = E\left(\frac{\partial\Psi(\alpha^*, \theta^*, \gamma, \beta_0)}{\partial\beta}\right), \qquad A_{\Psi_\alpha} = E\left(\frac{\partial\Psi(\alpha^*, \theta^*, \gamma, \beta_0)}{\partial\alpha}\right), \qquad B_Q = E\left(\frac{\partial Q(\alpha^*)}{\partial\alpha}\right)$$

$$A_{\Psi_\theta} = E\left(\frac{\partial\Psi(\alpha^*, \theta^*, \gamma, \beta_0)}{\partial\theta}\right), \qquad B_M = E\left(\frac{\partial M(\theta^*)}{\partial\theta}\right)$$

Moreover, assuming that the selection bias function and γ are correctly chosen, then

(1) when the dropout models are correctly specified, even if the regression models are not, we can replace Ω_Ψ in (G.1) by $E\left[\left\{\Psi(\alpha^*, \theta^*, \gamma, \beta_0) - A_{\Psi_\alpha}B_Q^{-1}Q(\alpha^*)\right\}^{\otimes 2}\right]$,

(2) when the regression models are correctly specified, even if the dropout models are not, we can replace Ω_Ψ in (G.1) by $E\left[\left\{\Psi(\alpha^*, \theta^*, \gamma, \beta_0) - A_{\Psi_\theta}B_M^{-1}M(\theta^*)\right\}^{\otimes 2}\right]$, and

(3) when both the dropout and the regression models are correctly specified, we can replace Ω_Ψ in (G.1) by $E\left[\left\{\Psi(\alpha^*, \theta^*, \gamma, \beta_0)\right\}^{\otimes 2}\right]$.

Each component of the asymptotic variance can be then estimated from their corresponding sample averages, with $(\alpha^*, \theta^*, \beta_0)$ replaced with $(\hat{\alpha}, \hat{\theta}, \hat{\beta})$.

Note that if the dropout and regression models are misspecified, the variance estimator is still consistent (i.e. expression G.1 still holds with α^*, θ^* and β_0 replaced by their probability

limits), even though the point estimator $\hat{\beta}$ is, in general, not consistent. Hence, to ensure double-robustness of the variance estimator, we recommend estimating variance using (G.1).

Proof. We provide a sketch proof. Let $U(\alpha^*, \theta^*, \gamma, \beta_0) = \Psi(\alpha^*, \theta^*, \gamma, \beta_0) - A_{\Psi_\alpha} B_Q^{-1} Q(\alpha^*) - A_{\Psi_\theta} B_M^{-1} M(\theta^*)$. We assume that regularity conditions 1-9 of Appendix B of Robins *and others* (1994) hold. But we replace $U(\alpha^*, \theta^*, \gamma, \beta_0)$ and $(\alpha^*, \theta^*, \beta_0)$ for their $H_i(\gamma)$ and γ_0, respectively, and replace $P(R_t = 1 | \bar{O}_{t-1}, Y_t, A_t = 1; \alpha) > c > 0$, $\forall \alpha$ and some c for their regularity assumption 3. We can expand equations (E.1) around β_0, then around α^* and around θ^*. This will give us:

$$\sqrt{n}(\hat{\beta} - \beta_0) = n^{\frac{1}{2}} \sum_{i=1}^{n} \left[-E\left(\frac{\partial \Psi(\alpha^*, \theta^*, \beta_0)}{\partial \beta}\right)^{-1} \left\{ \Psi_i(\alpha^*, \theta^*, \beta_0) - E\left(\frac{\partial \Psi(\alpha^*, \theta^*, \beta_0)}{\partial \alpha}\right) E\left(\frac{\partial Q(\alpha^*)}{\partial \alpha}\right)^{-1} Q_i(\alpha^*) - E\left(\frac{\partial \Psi(\alpha^*, \theta^*, \beta_0)}{\partial \theta}\right) E\left(\frac{\partial M(\theta^*)}{\partial \theta}\right)^{-1} M_i(\alpha^*) \right\} \right]$$

In Web Appendix E, we showed that the AIPW estimating equations are unbiased when mortal-cohort NFD holds, the selection bias function and the sensitivity parameter γ are correctly chosen, and if either the dropout models are correctly specified at all time points or the regression models are correctly specified at all time points.

Suppose that the dropout models are correctly specified, but the conditional mean outcome regression models are not (for all t). This implies that $\alpha^* = \alpha_0$, but $\theta^* \neq \theta_0$. Using equation (E.1), it is not difficult to show that $A_{\Psi_\theta} = 0$, and the results from (1) follows. Similarly, if the dropout models are correctly specified, but the conditional mean outcome regression models are not (for all t), then $\alpha^* = \alpha_0$ and $\theta^* \neq \theta_0$. Using equation (E.2), it is not difficult to show that $A_{\Psi_\alpha} = 0$, and the results from (2) follows.

When $\alpha^* = \alpha_0$ and $\theta^* = \theta_0$ (i.e. both of the dropout and the conditional mean outcome regression models are correctly specified for all t), it can be shown that $A_{\Psi_\alpha} = 0$ and $A_{\Psi_\theta} = 0$, and the results from (3) follows.

Appendix H

Mapping of assumptions to MAR

Wen *and others* (2017) described three assumptions related to MAR for monotone missing data. These assumptions are called unconditional MAR (u-MAR), partly conditional MAR (p-MAR) and fully conditional MAR (f-MAR). u-MAR is the assumption that

$$P(R_t = 1 | R_{t-1} = 1, \bar{Y}_s, D = s, X) = P(R_t = 1 | R_{t-1} = 1, \bar{Y}_{t-1}, A_t = 1, X) \quad \forall t \leqslant s$$

p-MAR is the assumption that

$$P(R_t = 1 | R_{t-1} = 1, \bar{Y}_s, A_s = 1, X) = P(R_t = 1 | R_{t-1} = 1, \bar{Y}_{t-1}, A_s = 1, X) \quad \forall t \leqslant s$$

and f-MAR is the assumption that

$$P(R_t = 1 | R_{t-1} = 1, \bar{Y}_s, D = s, X) = P(R_t = 1 | R_{t-1} = 1, \bar{Y}_{t-1}, D = s, X) \quad \forall t \leqslant s$$

As stated in Wen *and others* (2017), u-MAR implies p-MAR and f-MAR, but neither p-MAR nor f-MAR implies the other.

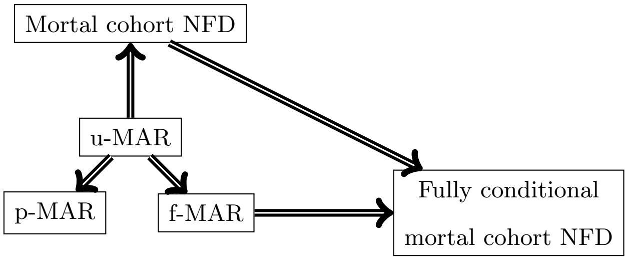

Figure 1. Relationship between assumptions (arrows represent implied relationships)

The figure above shows, for monotone missing data, the relationship between u-MAR and f-MAR, and mortal-cohort NFD and fully conditional mortal-cohort NFD of the current paper. By definition, the data $(X, Y_1, \ldots, Y_J)$ are MAR if $P(\bar{R}_t = \bar{r}_t \mid \bar{Y}_t, X) = P(\bar{R}_t = \bar{r}_t \mid \bar{Y}_{t,\text{obs}}(\bar{r}_t), X)$ for all t and $\bar{r}_t$, where $\bar{Y}_{t,\text{obs}}(\bar{r}_t)$ denotes the elements of $\bar{Y}_t$ that are observed when $\bar{R}_t = \bar{r}_t$. It can be shown that if $(X, Y_1, \ldots, Y_D)$ are MAR conditional on D, then the

data are f-MAR. Similarly if, for all t, $(X, Y_1, \ldots, Y_t)$ are MAR conditional on $\{A_t = 1\}$, then the data are p-MAR.

By definition, for monotone missing data, the data $(X, Y_1, \ldots, Y_J)$ are MNAR if $P(\bar{R}_t = \bar{r}_t \mid \bar{Y}_t, X) \neq P(\bar{R}_t = \bar{r}_t \mid \bar{Y}_{t,\text{obs}}(\bar{r}_t), X)$ for some t and $\bar{r}_t$. If $(X, Y_1, \ldots, Y_D)$ are MNAR conditional on D, then R_t could depend on future outcomes $Y_t \ldots Y_D$, which is a weaker assumption than fully-conditional mortal-cohort NFD. Similarly, if $(X, Y_1, \ldots, Y_t)$ are MNAR conditional on $\{A_t = 1\}$, then R_t could depend on future outcomes, which is a weaker assumption than mortal-cohort NFD.

With non-monotone missingness, sequential explainability is not the same as MAR. Sequential explainability says nothing about dependence of R_t on outcomes, whereas under MAR, R_t could depend on observed future outcomes. Hence, with non-monotone missingness, our assumptions have no immediate connection to MAR (or MNAR).

Appendix I

Supplementary results from HRS data

To better understand the reasons for the associations seen in the partly conditional model for the HRS data, we conducted a survival analysis where the event is death. Time to death, T, equals j if the subject died in the jth interval, i.e. if the subject died in $[t_j, t_{j+1})$ $(j = 1, 2, \ldots, 5)$, and equals 6 if the subject was still alive after the 5th interval. In particular, the first interval ($j = 1$) is $[0, 2)$, second interval ($j = 2$) is $[2, 4)$, third interval ($j = 3$) is $[4, 6)$, fourth interval ($j = 4$) is $[6, 8)$ and fifth interval ($j = 5$) is $[8, 10)$ years after baseline.

$$\text{logit}P(T = j \mid T \geqslant j, X) = \alpha_0 + \sum_{k=2}^{5} \alpha_l I(j = k) + \alpha_5\text{age} + \alpha_6\text{sex} + \alpha_7\text{edu} + \alpha_8 Y_{j-1} + \alpha_9 Y_{j-1}\text{age} + \alpha_{10} Y_{j-1}\text{sex} + \alpha_{11} Y_{j-1}\text{edu} + \alpha_{12}\text{age}\cdot\text{sex} + \alpha_{13}\text{age}\cdot\text{edu} + \alpha_{14}\text{sex}\cdot\text{edu}$$

where sex=1 if female and 0 if male, 'edu' is years of education, 'age' is recruitment age (centred at 80), and Y_{j-1} is the most recent cognitive score (centred at 18). We performed survival analyses for imputed outcomes under $\gamma = \{-0.2, -0.25, -0.3\}$ and found that results did not differ much from the three datasets. Parameter estimates (empirical standard errors using bootstrap) are given in the tables below:

γ	int.	$I(j=2)$	$I(j=3)$	$I(j=4)$	$I(j=5)$	age	sex	edu
-0.20	-2.128 (0.207)	0.220 (0.081)	0.808 (0.082)	0.437 (0.090)	0.488 (0.108)	0.101 (0.026)	-0.590 (0.229)	0.009 (0.016)
-0.25	-2.117 (0.207)	0.223 (0.081)	0.808 (0.082)	0.443 (0.090)	0.493 (0.107)	0.100 (0.026)	-0.580 (0.230)	0.007 (0.015)
-0.30	-2.100 (0.207)	0.226 (0.081)	0.812 (0.082)	0.451 (0.090)	0.505 (0.107)	0.099 (0.026)	-0.572 (0.229)	0.005 (0.015)

γ	Y_{j-1}	Y_{j-1}age	Y_{j-1}sex	Y_{j-1}edu	age*sex	age*edu	sex*edu
-0.20	-0.060 (0.018)	0.0015 (0.0012)	0.003 (0.010)	-0.003 (0.001)	-0.014 (0.014)	0.000 (0.002)	0.020 (0.017)
-0.25	-0.058 (0.018)	0.0014 (0.0012)	0.004 (0.009)	-0.003 (0.001)	-0.014 (0.014)	0.001 (0.002)	0.019 (0.017)
-0.30	-0.056 (0.018)	0.0012 (0.0011)	0.004 (0.009)	-0.003 (0.001)	-0.014 (0.014)	0.001 (0.002)	0.019 (0.017)

If the association between being a woman and a faster decrease over time in mean outcome given survival is partly due to mortality being higher in women with good cognitive function than in men with good cognitive function, then we would expect to see a positive value for α_{10}

(the parameter associated with Y_{j-1}sex). However, results show that α_{10} is not statistically significant ($\exp(\alpha_{10}) = 1.004, p = 0.70$).

Similarly, if the association between being older and a faster decrease over time in mean outcome given survival is partly due to mortality being higher in older subjects with good cognitive function than in younger subjects with good cognitive function, then we would expect to see a positive value for α_9 (the parameter associated with Y_{j-1}age). However, results show that α_9 is not statistically significant ($\exp(\alpha_9) = 1.001, p = 0.24$).

[Table 2 about here.]

[Table 3 about here.]

References

Chiba, Y. (2012). The large sample bounds on the principal strata effect with application to a prostate cancer prevention trial. *The International Journal of Biostatistics* **8**.

Chiba, Y. and VanderWeele, T. J. (2011). A simple method for principal strata effects when the outcome has been truncated due to death. *American Journal of Epidemiology* **173**, 745–751.

Ding, P., Geng, Z., Yan, W. and Zhou, X. (2011). Identifiability and estimation of causal effects by principal stratification with outcomes truncated by death. *Journal of the American Statistical Association* **106**, 1578–1591.

Egleston, B. L., Scharfstein, D. O., Freeman, E. E. and West, S. K. (2007). Causal inference for non-mortality outcomes in the presence of death. *Biostatistics* **8**, 526–545.

Egleston, B. L., Scharfstein, D. O. and MacKenzie, E. (2009). On estimation of the survivor average causal effect in observational studies when important confounders are missing due to death. *Biometrics* **65**, 497–504.

Gilbert, P. B., Bosch, R. J. and Hudgens, M. G. (2003). Sensitivity analysis for the assessment of causal vaccine effects on viral load in hiv vaccine trials. *Biometrics* **59**, 531–541.

Hayden, D., Pauler, D. K. and Schoenfeld, D. (2005). An estimator for treatment comparisons among survivors in randomized trials. *Biometrics* **61**, 305–310.

Imai, K. (2008). Sharp bounds on the causal effects in randomized experiments with “truncation-by-death”. *Statistics & Probability Letters* **78**, 144–149.

Jemiai, Y., Rotnitzky, A., Shepherd, B. E. and Gilbert, P. B. (2007). Semiparametric estimation of treatment effects given base-line covariates on an outcome measured after a post-randomization event occurs. *Journal of the Royal Statistical Society: Series B* **69**, 879–901.

Kurland, B. F., Johnson, L. L., Egleston, B. L. and Diehr, P. H. (2009). Longitudinal data with follow-up truncated by death: Match the analysis method to research aims. *Statistical Science* **24**, 211–222.

Mattei, A. and Mealli, F. (2007). Application of the principal stratification approach to the faenza randomized experiment on breast self-examination. *Biometrics* **63**, 437–446.

Robins, J. M., Rotnitzky, A. and P., Zhao L. (1994). Estimation of regression coefficients when some regressors are not always observed. *Journal of American Statistical Association* **89**, 846–866.

Shepherd, B. E., Redman, M. W. and Ankerst, D. P. (2008). Does finasteride affect the severity of prostate cancer? a causal sensitivity analysis. *Journal of the American Statistical Association* **103**, 1392–1404.

Tsiatis, A. A. (2006). *Semiparametric Theory and Missing Data*, 1 edition. New York, NY: Springer.

Wen, L., Muniz-Terrera, G. and Seaman, S. R. (2017). Methods for handling

longitudinal outcome processes truncated by dropout and death. *Biostatistics*.

Yang, F. and Small, D. S. (2016). Using post-outcome measurement information in censoring-by-death problems. *Journal of the Royal Statistical Society: Series B* **78**, 299–318.

Zhang, J. L. and Rubin, D. B. (2003). Estimation of causal effects via principal stratification when some outcomes are truncated by death. *Journal of Educational and Behavioral Statistics* **28**, 353–368.

Zhang, J. L., Rubin, D. B. and Mealli, F. (2009). Likelihood-based analysis of causal effects of job-training programs using principal stratification. *Journal of the American Statistical Association* **104**, 166–176.

Table 1: Hypothetical study described in Section A.

Principal strata among the subjects in the hypothetical study

% subjects	Strata	Y(1)	A(1)	Y(0)	A(0)
50	S1	10	1	7	1
30	S2	2	1	$*$	0
20	S4	$*$	0	$*$	0

Principal strata among the subjects in the hypothetical study stratified by treatment or exposure (Z)

% subjects	Strata	E	Y(1)	A(1)	Y(0)	A(0)
25	S1	1	10	1	7	1
25	S1	0	10	1	7	1
15	S2	1	2	1	$*$	0
15	S2	0	2	1	$*$	0
10	S4	1	$*$	0	$*$	0
10	S4	0	$*$	0	$*$	0

Observed outcomes among subjects in the hypothetical study stratified by treatment or exposure (Z)

% subjects	E	A	Y
25	1	1	10
15	1	1	2
10	1	0	$*$
25	0	1	7
25	0	0	$*$

Table 2: Parameter estimate (standard error) from model (equation (1)) for cognitive function fitted to HRS data using the first selection bias function at more extreme γ values

	Intercept	t	t^2	Age	Sex	Edu	t·Age	t·Sex	t·Edu
γ					IPW				
-0.4	11.366 (0.500)	-0.291 (0.134)	-0.012 (0.011)	-0.330 (0.031)	0.131 (0.259)	0.699 (0.037)	-0.016 (0.011)	-0.221 (0.068)	-0.008 (0.008)
-0.5	11.348 (0.523)	-0.338 (0.141)	-0.010 (0.011)	-0.330 (0.031)	0.188 (0.273)	0.689 (0.039)	-0.015 (0.011)	-0.229 (0.072)	-0.007 (0.009)
-0.6	11.355 (0.542)	-0.380 (0.146)	-0.008 (0.011)	-0.332 (0.032)	0.224 (0.282)	0.683 (0.041)	-0.015 (0.012)	-0.231 (0.075)	-0.006 (0.009)
-0.7	11.371 (0.559)	-0.415 (0.150)	-0.007 (0.011)	-0.333 (0.033)	0.246 (0.289)	0.678 (0.042)	-0.014 (0.012)	-0.231 (0.077)	-0.004 (0.010)
					CMI				
-0.4	11.061 (0.468)	-0.246 (0.135)	-0.015 (0.011)	-0.321 (0.028)	-0.022 (0.233)	0.728 (0.033)	-0.019 (0.008)	-0.100 (0.062)	-0.017 (0.008)
-0.5	10.962 (0.473)	-0.288 (0.139)	-0.011 (0.011)	-0.323 (0.028)	-0.027 (0.237)	0.729 (0.033)	-0.019 (0.009)	-0.097 (0.064)	-0.018 (0.008)
-0.6	10.915 (0.478)	-0.327 (0.142)	-0.009 (0.012)	-0.325 (0.028)	-0.032 (0.240)	0.730 (0.034)	-0.020 (0.009)	-0.094 (0.065)	-0.018 (0.008)
-0.7	10.890 (0.481)	-0.354 (0.145)	-0.008 (0.012)	-0.326 (0.029)	-0.034 (0.242)	0.731 (0.034)	-0.020 (0.009)	-0.094 (0.066)	-0.017 (0.008)
					AIPW				
-0.4	11.022 (0.479)	-0.215 (0.132)	-0.013 (0.011)	-0.319 (0.028)	0.060 (0.239)	0.729 (0.034)	-0.020 (0.008)	-0.165 (0.060)	-0.016 (0.008)
-0.5	10.923 (0.485)	-0.227 (0.136)	-0.011 (0.011)	-0.317 (0.028)	0.069 (0.244)	0.727 (0.035)	-0.021 (0.009)	-0.170 (0.062)	-0.017 (0.008)
-0.6	10.866 (0.487)	-0.246 (0.138)	-0.009 (0.012)	-0.318 (0.029)	0.063 (0.245)	0.728 (0.035)	-0.021 (0.009)	-0.172 (0.063)	-0.018 (0.008)
-0.7	10.834 (0.488)	-0.267 (0.139)	-0.008 (0.012)	-0.320 (0.029)	0.050 (0.246)	0.730 (0.035)	-0.021 (0.009)	-0.170 (0.064)	-0.018 (0.008)

Table 3: Parameter estimate (standard error) from model (equation (1)) for cognitive function fitted to HRS data using the second selection bias function: $\gamma_1 = \{-0.20, -0.25, -0.30\}$, $\gamma_2 = c\gamma_1$, and $c = \{1.25, 1.5, 2\}$

γ_1	c	Intercept	t	t^2	Age	Sex	Edu	t·Age	t·Sex	t·Edu
						IPW				
-0.20	1.25	11.496 (0.465)	-0.243 (0.121)	-0.015 (0.010)	-0.331 (0.029)	0.053 (0.227)	0.713 (0.033)	-0.017 (0.010)	-0.211 (0.062)	-0.010 (0.008)
	1.50	11.503 (0.465)	-0.255 (0.121)	-0.015 (0.010)	-0.334 (0.029)	0.065 (0.228)	0.713 (0.033)	-0.015 (0.011)	-0.226 (0.063)	-0.009 (0.008)
	2.00	11.510 (0.466)	-0.272 (0.122)	-0.016 (0.010)	-0.338 (0.030)	0.085 (0.230)	0.713 (0.033)	-0.011 (0.011)	-0.250 (0.065)	-0.008 (0.008)
-0.25	1.25	11.562 (0.471)	-0.221 (0.123)	-0.015 (0.010)	-0.328 (0.029)	0.036 (0.235)	0.715 (0.034)	-0.019 (0.011)	-0.197 (0.064)	-0.010 (0.008)
	1.50	11.571 (0.471)	-0.236 (0.124)	-0.015 (0.010)	-0.331 (0.030)	0.045 (0.236)	0.715 (0.034)	-0.017 (0.011)	-0.212 (0.065)	-0.010 (0.008)
	2.00	11.580 (0.472)	-0.256 (0.125)	-0.016 (0.010)	-0.335 (0.031)	0.063 (0.238)	0.715 (0.034)	-0.014 (0.012)	-0.237 (0.067)	-0.009 (0.008)
-0.30	1.25	11.441 (0.479)	-0.262 (0.127)	-0.015 (0.010)	-0.332 (0.030)	0.080 (0.244)	0.710 (0.035)	-0.016 (0.011)	-0.222 (0.066)	-0.009 (0.008)
	1.50	11.446 (0.479)	-0.273 (0.127)	-0.015 (0.010)	-0.335 (0.030)	0.093 (0.245)	0.710 (0.035)	-0.013 (0.011)	-0.236 (0.067)	-0.008 (0.008)
	2.00	11.453 (0.479)	-0.289 (0.129)	-0.016 (0.010)	-0.340 (0.031)	0.115 (0.248)	0.710 (0.034)	-0.009 (0.012)	-0.258 (0.070)	-0.008 (0.008)
						CMI				
-0.20	1.25	11.519 (0.453)	-0.243 (0.125)	-0.018 (0.011)	-0.324 (0.027)	-0.021 (0.218)	0.720 (0.032)	-0.020 (0.008)	-0.090 (0.056)	-0.013 (0.008)
	1.50	11.523 (0.453)	-0.251 (0.125)	-0.019 (0.011)	-0.323 (0.027)	-0.018 (0.218)	0.719 (0.032)	-0.021 (0.008)	-0.092 (0.057)	-0.013 (0.008)
	2.00	11.526 (0.454)	-0.258 (0.127)	-0.022 (0.011)	-0.322 (0.027)	-0.012 (0.219)	0.717 (0.032)	-0.022 (0.008)	-0.095 (0.058)	-0.011 (0.008)
-0.25	1.25	11.384 (0.457)	-0.236 (0.128)	-0.019 (0.011)	-0.322 (0.027)	-0.020 (0.223)	0.722 (0.033)	-0.020 (0.008)	-0.097 (0.058)	-0.014 (0.008)
	1.50	11.387 (0.458)	-0.241 (0.129)	-0.020 (0.011)	-0.322 (0.027)	-0.015 (0.223)	0.721 (0.033)	-0.021 (0.008)	-0.098 (0.059)	-0.013 (0.008)
	2.00	11.388 (0.458)	-0.245 (0.130)	-0.023 (0.011)	-0.320 (0.027)	-0.010 (0.224)	0.720 (0.033)	-0.022 (0.009)	-0.100 (0.060)	-0.012 (0.008)
-0.30	1.25	11.256 (0.461)	-0.232 (0.131)	-0.019 (0.011)	-0.321 (0.027)	-0.016 (0.227)	0.724 (0.033)	-0.020 (0.008)	-0.100 (0.060)	-0.015 (0.008)
	1.50	11.257 (0.462)	-0.236 (0.132)	-0.020 (0.011)	-0.320 (0.027)	-0.012 (0.228)	0.723 (0.033)	-0.021 (0.008)	-0.101 (0.061)	-0.014 (0.008)
	2.00	11.257 (0.462)	-0.239 (0.133)	-0.023 (0.011)	-0.319 (0.027)	-0.006 (0.229)	0.722 (0.033)	-0.022 (0.009)	-0.103 (0.061)	-0.013 (0.008)
						AIPW				
-0.20	1.25	11.319 (0.455)	-0.232 (0.118)	-0.016 (0.010)	-0.326 (0.027)	0.027 (0.218)	0.728 (0.032)	-0.017 (0.008)	-0.146 (0.055)	-0.014 (0.007)
	1.50	11.344 (0.455)	-0.251 (0.119)	-0.016 (0.010)	-0.326 (0.027)	0.019 (0.218)	0.726 (0.032)	-0.017 (0.008)	-0.138 (0.055)	-0.013 (0.008)
	2.00	11.374 (0.456)	-0.270 (0.121)	-0.018 (0.011)	-0.326 (0.027)	0.006 (0.218)	0.724 (0.032)	-0.017 (0.008)	-0.125 (0.055)	-0.013 (0.008)
-0.25	1.25	11.433 (0.462)	-0.226 (0.122)	-0.015 (0.011)	-0.326 (0.027)	0.030 (0.224)	0.726 (0.033)	-0.017 (0.008)	-0.146 (0.056)	-0.013 (0.008)
	1.50	11.465 (0.462)	-0.250 (0.123)	-0.016 (0.011)	-0.327 (0.027)	0.024 (0.224)	0.723 (0.033)	-0.016 (0.008)	-0.140 (0.056)	-0.012 (0.008)
	2.00	11.507 (0.462)	-0.279 (0.125)	-0.017 (0.011)	-0.327 (0.027)	0.010 (0.225)	0.721 (0.033)	-0.016 (0.008)	-0.127 (0.057)	-0.012 (0.008)
-0.30	1.25	11.210 (0.468)	-0.231 (0.126)	-0.015 (0.011)	-0.324 (0.028)	0.031 (0.230)	0.729 (0.033)	-0.018 (0.008)	-0.148 (0.058)	-0.015 (0.008)
	1.50	11.229 (0.469)	-0.245 (0.127)	-0.017 (0.011)	-0.324 (0.028)	0.023 (0.231)	0.727 (0.033)	-0.018 (0.008)	-0.140 (0.058)	-0.014 (0.008)
	2.00	11.249 (0.468)	-0.258 (0.128)	-0.019 (0.011)	-0.323 (0.028)	0.012 (0.231)	0.726 (0.033)	-0.018 (0.008)	-0.128 (0.059)	-0.014 (0.008)
